# Activation-induced thrombospondin-4 works with thrombospondin-1 to build cytotoxic supramolecular attack particles

**DOI:** 10.1073/pnas.2413866122

**Published:** 2025-02-04

**Authors:** Chiara Cassioli, Nagaja Capitani, Claire C. Staton, Claudia Schirra, Francesca Finetti, Anna Onnis, Nadia Alawar, Szu-Min Tu, Ludovica Lopresti, Vanessa Tatangelo, Carmela Tangredi, Salvatore Valvo, Hsin-Fang Chang, Annachiara Miccoli, Ewoud B. Compeer, Jemma Nicholls, Bruce R. Blazar, Giuseppe Marotta, Matthew J. A. Wood, Livio Trentin, Laura Patrussi, Michael L. Dustin, Ute Becherer, Cosima T. Baldari

**Affiliations:** ^a^Department of Life Sciences, University of Siena, Siena 53100, Italy; ^b^Kennedy Institute of Rheumatology, Nuffield Department of Orthopaedics, Rheumatology and Musculoskeletal Sciences, University of Oxford, Oxford OX3 7FY, United Kingdom; ^c^Institute for Developmental and Regenerative Medicine, Department of Paediatrics, University of Oxford, Oxford OX3 7TY, United Kingdom; ^d^Department of Cellular Neurophysiology, Center for Integrative Physiology and Molecular Medicine, Saarland University, Homburg 66421, Germany; ^e^Department of Pediatrics, Division of Blood & Marrow Transplant & Cellular Therapy, University of Minnesota, Minneapolis, MN 55455; ^f^Stem Cell Transplant and Cellular Therapy Unit, Siena University Hospital, Siena 53100, Italy; ^g^Hematology Unit, Department of Medicine, University of Padua, Padua 35128, Italy

**Keywords:** cytotoxicity, immunology, secretion, microscopy, leukemia

## Abstract

Cytotoxic attack particles released by CTLs and NK cells include diverse phospholipid membrane and glycoprotein encapsulated entities that contribute to target cell killing. Supramolecular attack particles (SMAPs) are one type of particle characterized by a cytotoxic core enriched in granzymes and perforin surrounded by a proteinaceous shell including thrombospondin (TSP)-1. TSP-4 was also detected in bulk analysis of SMAPs released by CTLs; however, it has not been investigated whether TSP-4 contributes to distinct SMAP types or the same SMAP type as TSP-1 and, if in the same type of SMAP, whether TSP-4 and TSP-1 cooperate or compete. Here, we observed that TSP-4 expression increased upon CD8^+^ T cell activation while, surprisingly, TSP-1 was down-regulated. Correlative Light and Electron Microscopy and Stimulated Emission Depletion microscopy localized TSP-4 and TSP-1 in SMAP-containing multicore granules. Superresolution dSTORM revealed that TSP-4 and TSP-1 are usually enriched in the same SMAPs while particles with single-positive shells are rare. Retention Using Selective Hooks assays showed that TSP-4 localizes to the lytic granules faster than TSP-1 and promotes its accumulation therein. TSP-4 contributed to direct CTL-mediated killing, as previously shown for TSP-1. TSP-4 and TSP-1 were both required for latent SMAP-mediated cell killing, in which released SMAPs kill targets after removal of the CTLs. Of note, we found that chronic lymphocytic leukemia (CLL) cell culture supernatants suppressed expression of TSP-4 in CTL and latent SMAP-mediated killing. These results identify TSP-4 as a functionally important component of SMAPs and suggest that SMAPs may be targeted for immune suppression by CLL.

## Significance

Cytotoxic T cells (CTLs) kill infected and cancer cells by introduction of granzymes into the cytoplasm of the target in a perforin-dependent manner. We have recently demonstrated that thrombospondin-1 (TSP-1) plays an important role in this process through encapsulating half of the released granzymes and perforin in supramolecular attack particles (SMAPs). Surprisingly, TSP-1 mRNA was down-regulated on T cell activation. In contrast, TSP-4 mRNA, which was not studied previously, was up-regulated. We demonstrate that TSP-1 and TSP-4 coassembled into the SMAPs and that they are both required for full CTL- and SMAP-mediated cytotoxicity. TSP-4 reaches granules in CTL faster than TSP-1. Chronic lymphocytic leukemia cells produce factors that impair expression of TSP-4, suggesting an immune evasion strategy.

Cytotoxic T cells (CTLs) eliminate virally infected and cancerous cells using a diversified arsenal of cytotoxic mediators stored in lysosome-related organelles that undergo exocytosis into the immunological synapse (IS) with a target cell (1, 2). Lytic granules (LGs) are specialized secretory lysosomes (3) that concentrate perforin (Prf), a pore-forming protein that enables a set of serine proteases, the granzymes (Gzm), to enter the cytoplasm of the target cell and rapidly trigger programmed cell death (1, 4, 5). Prf and Gzms are complexed with the proteoglycan serglycin (6–8) but disperse rapidly upon exocytosis into the IS and effectiveness is dependent upon confinement in the synaptic cleft. There are also diverse cytotoxic particles that are released from CTLs that contribute to killing in different scenarios. For example, CTLs can induce target cell death through Fas-ligand exposure on the T cell surface (9) or on extracellular vesicles (10, 11). Prf has also been found to be associated with extracellular vesicles (12). Prf and Gzm are encapsulated within a thrombospondin (TSP)-1 enriched glycoprotein shell in supramolecular attack particles (SMAPs) (13). SMAPs are stored in multicore granules (MCGs), a class of LGs differing from the canonical single-core LGs (SCGs) in size, morphology, and protein composition (2). SMAPs released into the extracellular space have latent activity and can kill targets that encounter the SMAPs after complete removal of CTLs (13), a process we define as latent killing. There are also a number of studies on cytotoxic particles from NK cells, which include extracellular vesicle fractions (14, 15) and SMAPs (16). We are only beginning to learn about the diversity of these killing entities and many questions remain.

A mass spectrometry-based analysis of SMAPs released by CTLs on planar supported lipid bilayer (PSLB) targets identified TSP-1 and TSP-4. Only TSP-1 was validated based on 1) finding the 60 kDa C-terminal fragment of TSP-1 in the shell of SMAPs and 2) CRISPR targeting of *THBS1* in CTLs impairing target killing (13). TSP-4 has not been investigated, but how different is TSP-4 from TSP-1? The TSPs are a family of evolutionarily conserved Ca^2+^-binding glycoproteins that undergo constitutive secretion to the extracellular matrix to regulate cell–cell and cell–matrix interactions (17–19). An exception to this is TSP-1 in platelets, which store TSP-1 in α-granules and release it on activation (20). Importantly, TSP-1 and TSP-4 are members of different branches of the TSP family. The human TSP family consists of five members that share a C-terminal signature domain, comprising a series of EGF-like domains and type 3 repeats that bind up to 30 Ca^2+^ ions and a C-terminal lectin-like domain. The N terminus of TSPs is more variable and differs in domain organization and degree of oligomerization. Group A TSPs are trimeric and include TSP-1 and TSP-2, whereas group B members are pentameric and include TSP-3, TSP-4, and TSP-5/cartilage oligomeric matrix protein (21–23). Additionally, group A TSPs are characterized by a N-terminal heparin-binding domain and central TSP type 1 domains, which are absent in group B TSPs (24). Biological roles of TSP-1 are mostly in the cardiovascular system, whereas TSP-4 is more associated with the musculoskeletal system (24). Our interest in cytotoxic particle heterogeneity led us to investigate TSP-4 further. Is TSP-4 a component of the same SMAPs as TSP-1 or another type of cytotoxic particle and, if present in the same SMAPs, does TSP-4 compete with or complement TSP-1 in SMAP biogenesis and function?

Here, we show that TSP-4 is a unique component of the SMAP shell that has distinct roles to TSP-1. First, TSP-4 expression is induced by activation of CD8^+^ T cells, whereas TSP-1 expression is decreased. Second, TSP-4 and TSP-1 colocalize in MCGs and both are components of the SMAP shell; however, TSP-4 reaches MCGs faster than TSP-1 and even promotes TSP-1 association with LGs. Third, TSP-4 participates in both CTL-mediated direct killing and SMAP-mediated latent killing, like TSP-1, but only TSP-4 expression was down-regulated by chronic lymphocytic leukemia (CLL) culture supernatants that suppress both direct killing by CTLs and latent killing by released SMAPs. These results provide insights into the process of SMAP biogenesis through differential regulation of TSP expression and TSP cooperation.

## Results

### Complementary Profiles of TSP-1 and TSP-4 Expression during CD8^+^ T Cell Differentiation to CTLs.

**Fig. 1.**
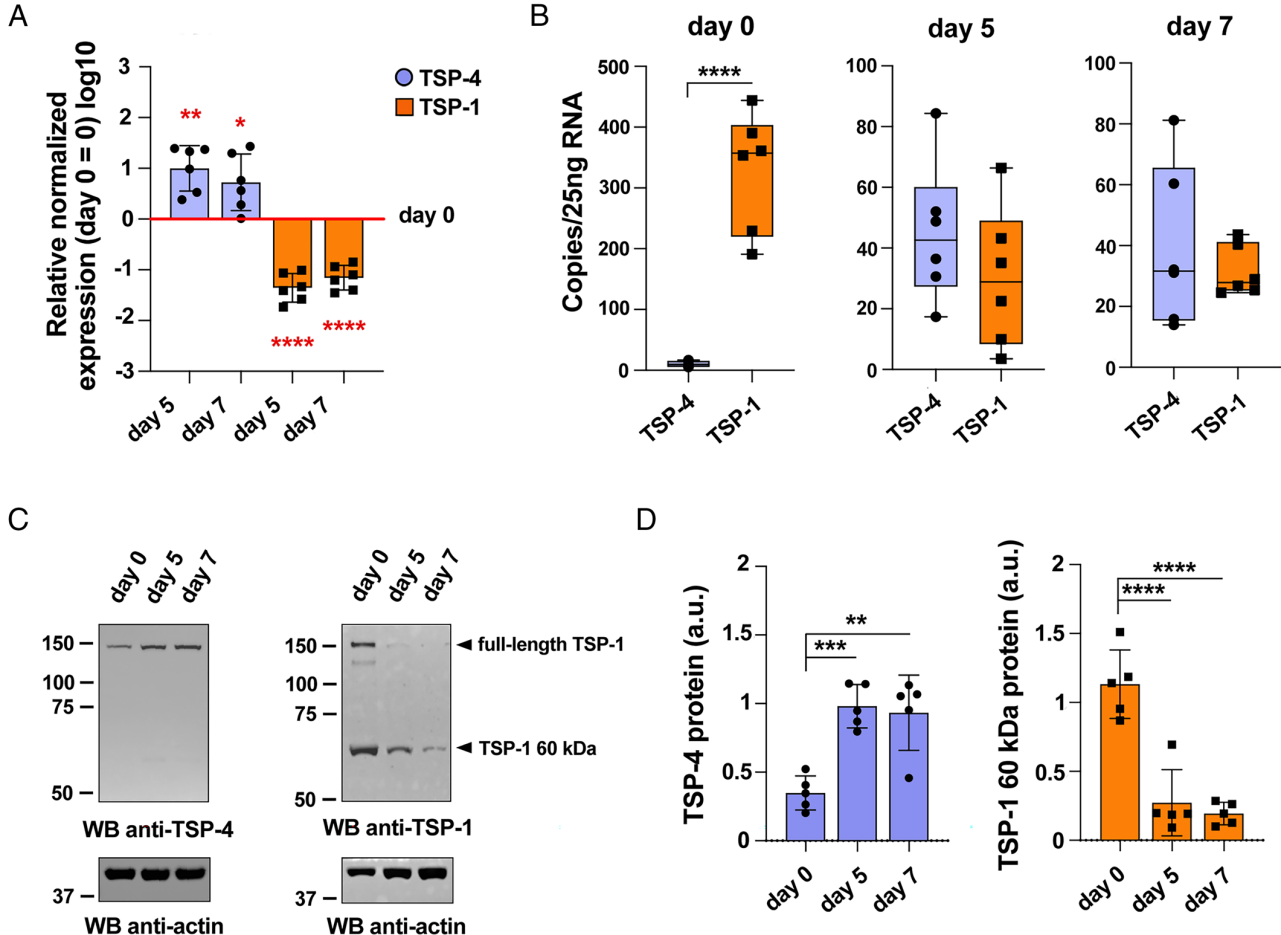
TSP-1 and TSP-4 show an opposite expression profile during CD8^+^ T cell differentiation to CTLs. (*A*) Time course analysis of TSP-4 and TSP-1 mRNA levels in freshly isolated CD8^+^ T cells (day 0) and 5- and 7-d CTLs. The graph shows the normalized relative abundance (mean ± SD, ctr value = 1) of TSP-4 and TSP-1 transcripts. N_donors_ = 6, one-way ANOVA test; ****P ≤ 0.0001, **P ≤ 0.01, *P ≤ 0.05, only significant differences are shown. (*B*) Absolute quantification of TSP-4 and TSP-1 copies (mean ± SD) in freshly isolated CD8^+^ T cells (day 0) and 5- and 7-d CTLs by droplet digital PCR. N_donors_ = 6, unpaired *t* test; ****P ≤ 0.0001, only significant differences are shown. (*C* and *D*) Immunoblot analysis of TSP-4 (*Left*) and TSP-1 (*Right*) in lysates of freshly isolated CD8^+^ T cells (day 0) and 5- and 7-d CTLs. Arrowheads in (*Right*) indicate full-length TSP-1 and its 60 kDa species. Quantification (mean ± SD) of the relative TSP-4 and 60 kDa TSP-1 expression normalized to actin used as loading control is shown in panel *D*. The migration of molecular mass markers is indicated (kDa). N_donors_ = 5, one-way ANOVA test; ****P ≤ 0.0001, ***P ≤ 0.001, **P ≤ 0.01, only significant differences are shown.

To investigate TSP-4 expression in human CD8^+^ T cells and its regulation during their differentiation to CTLs, we first established a protocol for in vitro generation of CTLs. Total CD8^+^ T cells purified by negative selection from buffy coats of healthy donors were activated with magnetic beads coated with anti-CD3 and anti-CD28 antibodies in the presence of IL-2 and further expanded for 3 to 5 d after activation (*SI Appendix*, Fig. S1*A*), when they expressed the typical markers of effector T cells (*SI Appendix*, Fig. S1*B*) and acquired cytolytic activity as assessed by GzmB and Prf expression (*SI Appendix*, Fig. S1*C*). Killing assays performed using MEC1 cells loaded with a mix of Staphylococcal superantigens (SAgs) for polyclonal activation confirmed that these CTLs were able to effectively kill target cells (*SI Appendix*, Fig. S1 *D*, E). Expression of TSP-4 (encoded by *THBS4*) during CTL differentiation was compared to that of TSP-1 (encoded by *THBS1*) by RT-qPCR analysis of mRNA purified from total CD8^+^ T cells either freshly isolated (time 0) or at days 3 and 5 after activation (days 5 and 7; see *SI Appendix*, Fig. S1*A*). Surprisingly, TSP-1 and TSP-4 expression underwent a differentiation-related regulation in opposite directions. While detectable at relatively low levels in freshly isolated CD8^+^ T cells, TSP-4 was upregulated during their differentiation to CTLs (Fig. 1*A*). Conversely, TSP-1 was expressed at high levels in freshly purified total CD8^+^ T cells followed by a substantial decrease upon initiation of activation and differentiation to CTL (Fig. 1*A*), although TSP-1 transcripts were detectable at all stages. To directly compare TSP-1 vs. TSP-4 expression we quantified the respective transcripts in the same reaction using droplet digital PCR. While at day 0 the number of TSP-1 transcripts was substantially higher than TSP-4, there was no statistically significant difference at days 5 and 7, indicating that *THBS1* and *THBS4* are expressed at comparable levels in differentiated CTLs (Fig. 1*B*). This analysis also confirmed the differentiation-related changes in *THBS1* and *THBS4* expression. The opposite expression pattern of TSP-1 and TSP-4 was also observed at the protein level by immunoblot. Freshly purified CD8^+^ T cells showed low levels of a ~150 kDa immunoreactive TSP-4 species that increased in CTLs (Fig. 1 *C* and *D*). Conversely, freshly purified CD8^+^ T cells showed high levels of TSP-1, with a prevalence of the 60 kDa isoform reported to be associated with SMAPs (13) that dropped during differentiation to CTLs, although TSP-1 remained detectable at all timepoints analyzed (Fig. 1 *B* and *C*). The specificity of the TSP-1 and TSP-4 immunoreactive bands was confirmed in TSP-1/TSP-4 KO CTLs (*SI Appendix*, Fig. S1 *F* and G). These results demonstrate that TSP-4 is expressed in CTLs, validating the mass spectrometry data (13) and, surprisingly, show that expression of TSP-1 and TSP-4 is modulated in opposite directions during CTL differentiation.

### TSP-4 Colocalizes with TSP-1 in MCGs in CTLs.

To analyze the intracellular localization of TSP-4 in CTLs, we generated a construct encoding mCherry-tagged TSP-4 (*SI Appendix*, Fig. S2*A*). Flow cytometric and immunoblot analysis of TSP-4 in CTLs transiently transfected with either the mCherry-encoding vector or the TSP-4-mCherry construct confirmed the expression of recombinant TSP-4-mCherry (*SI Appendix*, Fig. S2 *B* and C). Additionally, fluorescence microscopy analysis of the CTL transfectants showed a punctate staining for TSP-4-mCherry, as opposed to the diffuse cytoplasmic staining of mCherry (*SI Appendix*, Fig. S2*D*). For comparison, the same analysis was carried out for TSP-1-GFPSpark and TSP-4-GFPSpark, using fluorescently tagged TSPs or the respective GFPSpark control (*SI Appendix*, Fig. S2 *A*, E, and *F*). The results confirmed the granular pattern of TSP-1-GFPSpark in CTLs (*SI Appendix*, Fig. S2*G*) (13). Staining with anti-TSP-1/4 antibodies confirmed the recombinant fluorescent TSPs as appropriate readouts for their endogenous counterparts (*SI Appendix*, Fig. S2 *H* and I).

To determine the subcellular localization of TSP-4 within the cytolytic endosomal compartment, we carried out a confocal immunofluorescence analysis of CTLs transiently transfected with mCherry-tagged TSP-4 and costained for the LG markers GzmB, Prf, and LAMP-1. The cis-Golgi marker GM130 was used as a negative control. Colocalization analyses showed that TSP-4-mCherry was associated with GzmB, Prf, and LAMP-1 positive LGs (*SI Appendix*, Fig. S3*A*). A similar analysis carried out on TSP-1-GFPSpark expressing CTLs (*SI Appendix*, Fig. S3*B*) confirmed the LG localization of TSP-1 (13).

While the mass spectrometry analysis of CTL-derived SMAPs revealed the presence of both TSP-1 and TSP-4 (13), whether they colocalize within the same LGs is not known. To address this issue, we cotransfected CTLs with the constructs encoding TSP-4-mCherry and TSP-1-GFPSpark. TSP-4 showed a significant vesicular colocalization with TSP-1 (*SI Appendix*, Fig. S3*C*). Accordingly, a substantial subpopulation of LGs contains both TSP-4 and TSP-1 (*SI Appendix*, Fig. S3*C*). Additionally, TSP-1 and TSP-4 participate in the same complexes, as assessed by a coimmunoprecipitation analysis of CTLs cotransfected with the constructs encoding TSP-4-mCherry and TSP-1-GFPSpark (*SI Appendix*, Fig. S3*D*).

SMAPs are stored in a unique LG population, the MCGs, that can be identified by their unique components TSP-1 and WGA (2). The vesicular colocalization of TSP-4 with TSP-1 suggests that, similar to TSP-1, TSP-4 may be selectively directed to the MCGs. However, the limited resolution of confocal microscopy could lead to false positive results for colocalization and led us to extend these studies to superresolution approaches. We first performed a Correlative Light and Electron Microscopy (CLEM) analysis of CTLs coexpressing TSP-4-mCherry and TSP-1-GFPSpark. 16 h posttransfection CTLs were incubated for 2 h with fluorescently tagged WGA to mark biosynthetically active MCGs that are receiving material from the endosomal pathway, then plated on sapphire disc-immobilized anti-CD3 Ab to induce IS formation and LG accumulation. Cells were subjected to high-pressure freezing, freeze-substituted, embedded in resin, sectioned into serial 100 nm sections and subjected to correlative high-resolution Structured Illumination Microscopy (SIM) and Transmission Electron Microscopy (TEM) analysis (*SI Appendix*, Fig. S4*A*). The fluorescent images were overlaid on the corresponding TEM images to visualize the ultrastructure of the TSP-1, TSP-4, and WGA fluorescent spots. Electron micrographs of human CTLs without electroporation are shown as control (*SI Appendix*, Fig. S4*B*).

**Fig. 2.**
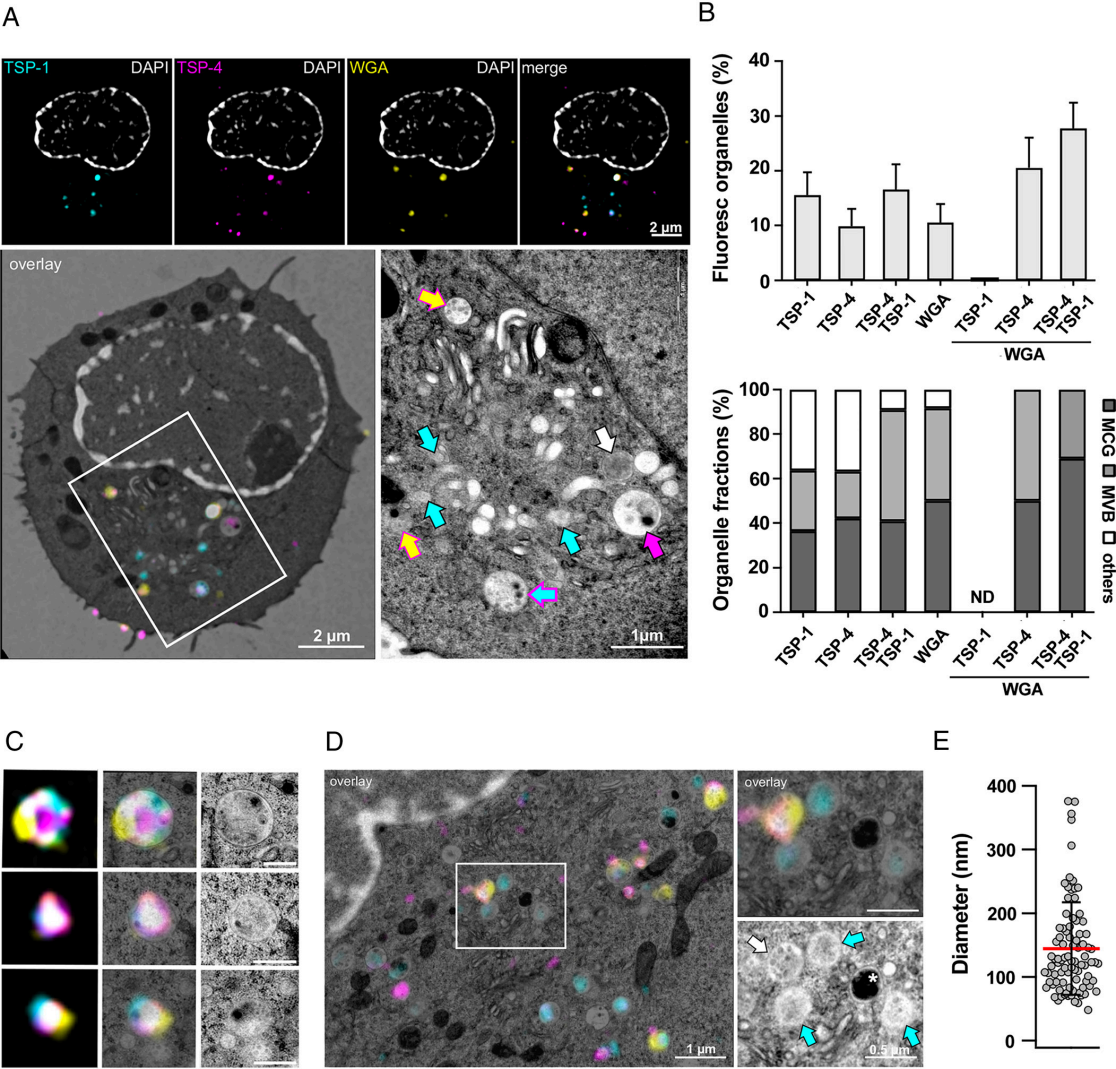
TSP-4, TSP-1, and WGA colocalize in MCGs. (*A*) CLEM image of a representative human CTL expressing TSP-1-GFPSpark and TSP-4-mCherry, preincubated with WGA-AF647, and stained with DAPI. The cell was stimulated by seeding it on an anti-CD3 (UCHT1) coated coverslip. Shown are SIM images (*Top* row) and the corresponding TEM overlay image (*Bottom* panel, *Left*). The white rectangle marks the region magnified in the *Lower*, *Right* image. (Scale bar, 2 μm.) In the magnified TEM image (*Right*), arrows point to different fluorescent proteins using the same color code as the *Upper* panels. (Scale bar, 1 μm.) (*B*) Quantification of the different fluorescent organelles present in the cell sections (*Upper* panel) defined by their expression profile (mean ± SEM). The lower panel shows the corresponding normalized organelle fractions according to their morphology (multicore granule, MCG; multivesicular body, MVB, and others). N_donors_ = 2, n_cells_ = 24, 137 organelles; ND, not detected. (*C*) Enlarged organelles which are positive for TSP-1-GFPspark, TSP-4-mCherry, and WGA-AF647 cropped from the images of three different human CTL. SIM images (*Left*), SIM/TEM overlay images (*Middle*), and TEM images (*Right*). (Scale bar, 0.5 μm.) (*D*) Representative CLEM image of a cell section as described in (*A*) showing a SIM image with the corresponding TEM image (*Left*). (Scale bar, 1 μm.) The white rectangle marks the region magnified in the *Right Upper* and *Lower* panels. The arrows point to different organelles according to the color code of the fluorescent proteins shown in (*A*). The white asterisk indicates a SCG. (Scale bar, 0.5 μm.) (*E*) Quantification of SMAP diameter observed in MCGs as shown in (*A* and *B*). N_donors_ = 2, n_cells_ = 24, 90 SMAPs. The red line represents mean and black lines SD.

The labeled organelles included populations that were either single positive or double positive for TSP-1 and TSP-4 (Fig. 2 *A*–*C*). Within this vesicle pool, our CLEM data demonstrate that the majority of TSP-1 and TSP-4 staining localizes to MCGs (Fig. 2 *B* and *C*), defined as the organelles that encapsulate multiple dense core particles identified as SMAPs (2). Consistent with their identity as SMAPs (13), the average diameter of densecore particles within the MCGs was 144.26 ±7.66 nm (Fig. 2*E*) (2). Interestingly, these were found to be either TSP-4^+^WGA^+^ or TSP-1^+^TSP-4^+^WGA^+^, while no TSP-1^+^WGA^+^ particles could be detected (Fig. 2*B*). This heterogeneity suggests a sequential transport of TSP-4 and TSP-1 to MCGs during their formation, with TSP-1 becoming incorporated at a later step of MCG biogenesis compared to TSP-4. Notably, similar to TSP-1 (2), we did not detect TSP-4 associated with single dense cores or compartmentalized within SCGs (Fig. 2*D*), indicating that TSP-4 does not localize to SCGs.

**Fig. 3.**
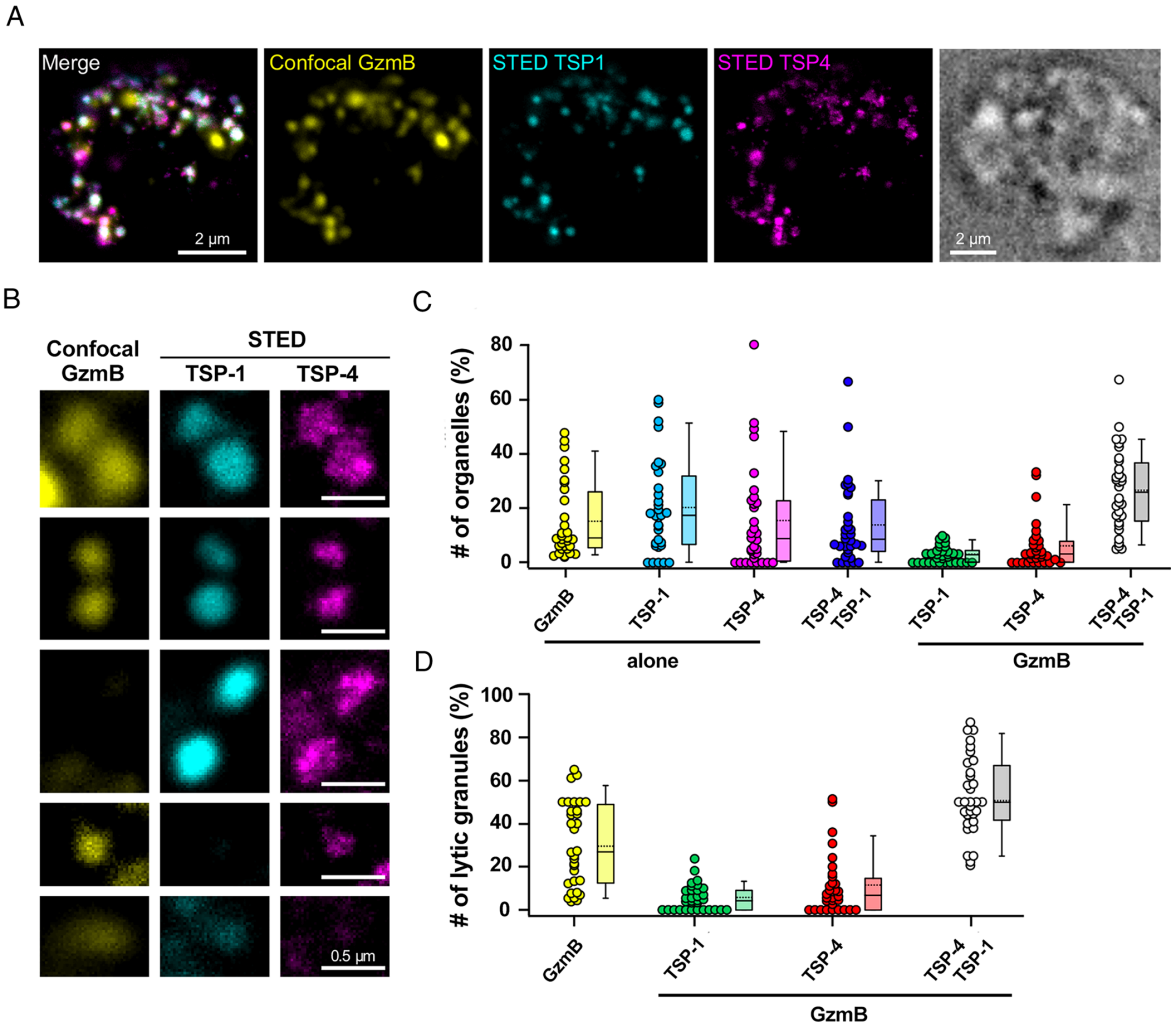
TSP-4 and TSP-1 localize predominantly together to LGs. (*A*) Representative stimulated human CTL expressing TSP-1-Flag (cyan) and TSP-4-HA (magenta) forming an IS with the anti-CD3 coated coverslip. Cells were fixed and stained with anti-GzmB (yellow), anti-Flag, and anti-HA antibodies. TSP-1 and TSP-4 were acquired in STED microscopy mode while GzmB was acquired in confocal mode. Shown are the merge image of all three channels and the images of the individual channels. On the *Right* is depicted the brightfield image of the same cell. (Scale bar, 2 µm.) (*B*) Enlarged pictures of organelles, which are positive for GzmB, TSP-1-Flag, and/or TSP-4-HA extracted from four different human CTL images as displayed in (*A*). (Scale bar, 0.5 µm.) (*C* and *D*) Object-based colocalization analysis of all three proteins displayed as a scatter dot plot with the values of individual cells superimposed with a box plot comprising the median (line) and the mean value (stippled line). Panel (*C*) shows the % of organelles with or without TSP-1 and/or TSP-4 among all individual organelles and panel (D) shows the % of LGs, i.e. organelles that display GzmB labeling, with or without TSP-1 and or TSP-4. N_donors_ = 2, n_cells_ = 32, 1,427 organelles.

To analyze the respective arrangement of TSP-4 and TSP-1 within MCGs, we exploited the superresolution capability of STimulated Emission Depletion (STED) microscopy. Day-6 CTLs were cotransfected with constructs encoding TSP-4-HA and TSP-1- FLAG. 16 h posttransfection they were plated on immobilized anti-CD3 Ab and costained with antibodies specific for HA, FLAG, and GzmB. GzmB was detected in confocal mode, while TSP-1-FLAG and TSP-4-HA were analyzed by STED microscopy. TSP-1 and TSP-4 showed a granular pattern (Fig. 3 *A* and *B*) and colocalized with GzmB in 26.54 ± 2.64 % (n = 32) of all granules (Fig. 3*C*). Among the LGs, identified as the granules positive for GzmB, the largest population was TSP-1^+^TSP-4^+^ (52.8 ± 3.2 %, n = 32), with a smaller population of TSP-4^+^GzmB^+^ granules and only a minor population of TSP-1^+^GzmB^+^ (Fig. 3*D*), consistent with the CLEM analysis, again suggesting an earlier incorporation of TSP-4 into MCGs compared to TSP-1.

### TSP-4 and TSP-1 Are Sequentially Transported to the LGs.

**Fig. 4.**
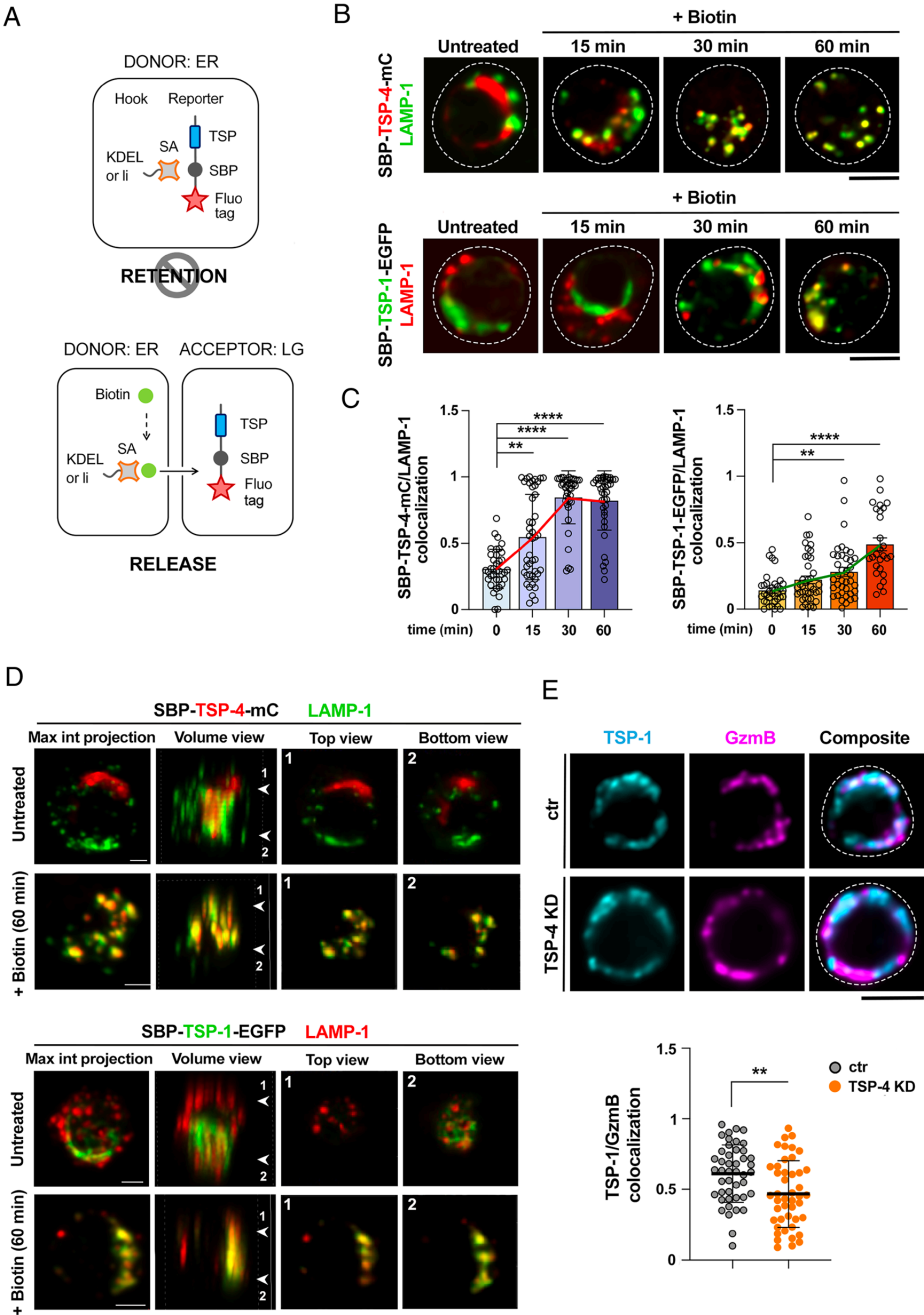
TSP-4 localizes to LGs prior to TSP-1 and promotes TSP-1 association with LGs. (*A*) Principle of the RUSH assay. In the absence of biotin, TSP-1- SBP-EGFP and TSP-4-SBP-mCherry reporters are retained in the ER by a streptavidin-li and a streptavidin-KDEL hook, respectively (*Left*). After biotin addition, TSP-1-SBP-EGFP and TSP-4-SBP-mCherry reporters leave the ER and traffic toward LAMP-1^+^ LGs (*Right*). (*B*–*D*). Time course analysis in CTLs expressing either Str- KDEL_SBP-TSP-4-mCherry (*Bottom*) or Str-li_SBP-TSP-1-EGFP (*Top*) in the absence or presence of biotin. Representative images (medial optical sections) are shown in (*B*). Dashed lines mark the cell outline. (Scale bar, 5 μm.) The histograms (*C*) show the quantification (mean ± SEM) of the weighted colocalization in medial optical sections using Manders’ overlap coefficient between TSP-4 (*Right*) or TSP-1 (*Left*) and LAMP-1 signals. N_donors_ = 2, n_cells_ > 20, Kruskal–Wallis test; ****P ≤ 0.0001, **P ≤ 0.01, only significant differences are shown. 3D view (from left to right: max intensity projection, volume, top and bottom slices) of representative CTLs expressing either Str- KDEL_SBP-TSP-4-mCherry (*Top*) or Str-li_SBP-TSP-1-EGFP (*Bottom*) in the absence or presence of biotin (60 min) are shown in (*D*). (Scale bar, 2 μm.) (*E*) *Top*, confocal images (medial optical sections) of CTLs cotransfected with TSP-1-GFPSpark and TSP-4-targeting siRNAs (TSP-4 KD) or control scrambled siRNAs. Dashed lines mark the cell outline. (Scale bar, 5 μm.) Quantification (mean ± SD) of the weighted colocalization using the Manders’ overlap coefficient between TSP-1 and GzmB signals. N_donors_ = 3, n_cells_ ≥ 40, unpaired *t* test; **P ≤ 0.01. *Bottom*, quantification (%) of vesicles single or double positive for TSP-1GFSpark and GzmB.

To elucidate whether TSP-4 localizes indeed to LGs prior to TSP-1, we used the Retention Using Selective Hooks (RUSH) assay (25). In this assay the protein of interest is tagged with a fluorescent tag and with streptavidin-binding protein (SBP), which allows its retention in a given membrane compartment through binding to a “hook”, an amino acid sequence from a protein resident in that specific compartment, tagged with streptavidin. On addition of biotin to the culture medium, the protein of interest is released from the donor compartment and can be tracked during its transport to its final compartment of destination (25). We generated two RUSH constructs, encoding either SBP-tagged TSP-1-EGFP or SBP-tagged TSP-4-mCherry, and functionally equivalent endoplasmic reticulum-specific hooks (li and KDEL, respectively) (Fig. 4*A*). These constructs were transfected by nucleofection into CTLs and tracked by confocal microscopy to their LAMP-1^+^ LG destination at different time points after addition of biotin. A time course analysis showed an increase in the colocalization of both TSPs with LAMP-1 after biotin addition with TSP-4 arriving prior to TSP-1 (Fig. 4 *B*–*D*), confirming the hypothesis that the two TSPs are sequentially transported to LGs.

The low abundance of TSP-1^+^ LGs compared to TSP-4^+^ and TSP-4^+^TSP-1^+^ ones (Figs. 2 and 3) raises the possibility that TSP-4 might facilitate the incorporation of TSP-1 into LGs. To test this hypothesis, we investigated the association of TSP-1 with LGs in ctr and TSP-4 KD CTLs that express comparable levels of TSP-1-GFPSpark. The colocalization of TSP-1 with GzmB was significantly decreased in TSP-4 deficient CTLs (Fig. 4*E*), indicating that TSP-4 not only localizes to LGs prior to TSP-1, but promotes TSP-1 incorporation into LGs.

### TSP-1 and TSP-4-Containing LGs Concentrate at the IS.

SMAPs are released at the IS formed by CTLs (13). To investigate the localization of TSP-4 with TSP-1 at the IS, we plated CTLs transfected with the TSP-1-GFPSpark and TSP-4-mCherry constructs on PSLBs functionalized with ICAM-1 to promote LFA-1-mediated adhesion, alone (nonactivating conditions) or in combination with an anti-CD3ε Fab′ (activating conditions) (*SI Appendix*, Fig. S5*A*). After 30 min incubation at 37 °C, cells were fixed and permeabilized, followed by staining with anti-GzmB antibodies to identify LGs. Consistent with the colocalization analysis of unstimulated CTLs (*SI Appendix*, Fig. S3), TSP-4-mCherry showed a significant colocalization with GzmB when plated on nonactivating PSLBs (*SI Appendix*, Figs. S5 *B* and C and S6 A and *B*), similar to TSP-1-GFPSpark (*SI Appendix*, Figs. S5 *B* and C and S6 A and *B*). Colocalization was enhanced under activating conditions, with an accumulation of TSP-4^+^GzmB^+^ vesicles and TSP-1^+^GzmB^+^ vesicles at the IS (*SI Appendix*, Figs. S5 *B* and C and S6 A and *B*). These vesicles correspond to MCGs, as shown by a strong colocalization of TSP-1 and TSP-4 with vesicles that are positive for the MCG marker WGA in CTLs plated on immobilized anti-CD3 mAb (*SI Appendix*, Fig. S5 *D*–F). Additionally, TSP-4 and TSP-1 colocalize under both homeostatic and activating conditions, even though the intracellular distribution of TSP-1^+^ and TSP-4^+^ LGs changed during activation from a dispersed within the cell in nonactivating conditions to a clustered at the IS in activating conditions (*SI Appendix*, Figs. S5 *B*–F and S6 A and *B*).

### TSP-1 and TSP-4 Are Coreleased in Association with SMAPs.

**Fig. 5.**
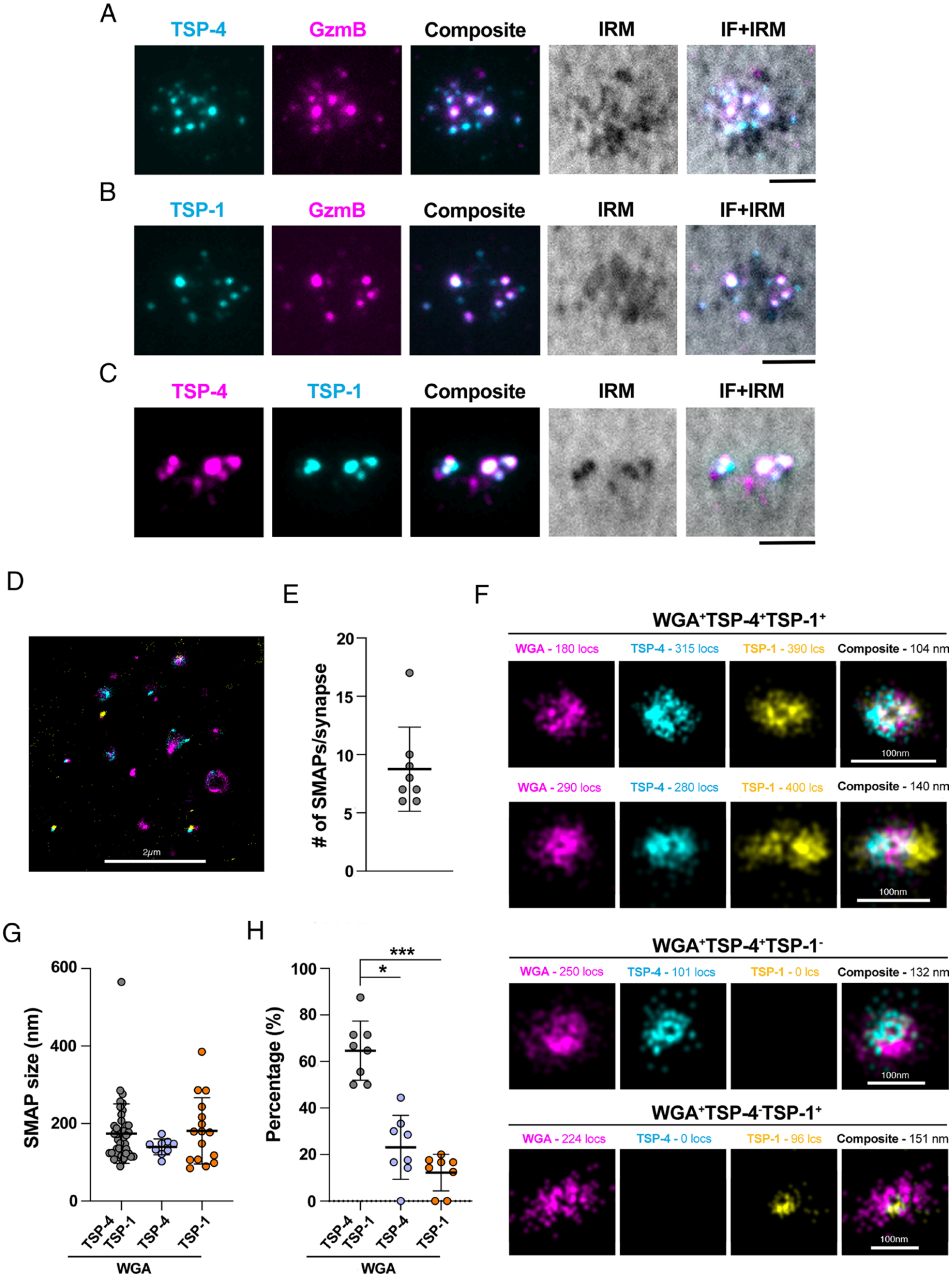
TSP-1 and TSP-4 are coreleased at the IS in association with SMAPs. (*A* and *B*) TIRF images of TSP-4-GFPSpark^+^ (cyan) and TSP-1-GFPSpark^+^ (cyan) particles released by CTLs coexpressing fluorescently tagged TSP-4 (*A*) or TSP-1 (*B*) and GzmB-mCherry on activating PSLBs [ICAM-1 + anti-CD3ε UCHT1 Fab′ (unlabeled)]. (*C*) TIRF images of TSP-4-mCherry^+^ (magenta) and TSP-1-GFPSpark^+^ (cyan) particles released by CTLs expressing fluorescently tagged TSP-1 or TSP-4 on activating PSLBs [ICAM-1 + anti-CD3ε UCHT1 Fab′ (unlabeled)]. N_donors_ = 3 donors, n_cells_ ≥ 10 cells. The nonfluorescent particles in the IRM images may correspond either to SMAPs released by nontransfected CTLs or other particles (e.g. exosomes) negative for GzmB, TSP-1, or TSP-4. IRM, interference reflection microscopy. (Scale bar, 2.5 μm.) (*D*) Analysis of colocalization of TSP-1 and TSP-4 in SMAPs released by CTLs using 2D SMLM dSTORM to achieve single particle, single molecule resolution. The micrograph shows SMAPs deposited following IS formation of CTLs on the activating PSLBs. The complete c-SMAC region is shown. (Scale bar, 2 µm.) (*E*) Average number of SMAPs per IS depicted as a box plot including outliers. (*F*) Representative micrographs of single particles in CTL derived ISs. SMAPs are stained with WGA-AF647, TSP4-mCherry was probed with anti-RFP-AF555 and TSP1-GFPSpark was probed with anti-GFP-AF488. (*G*) Average size of SMAPs in each category depicted as a box plot including outliers. Kruskal–Wallis test; only significant differences are shown. (*H*) Percentage of SMAPs positive for WGA, TSP1, and TSP4 derived from CTLs of two healthy donors (8 ISs and n = 70 particles). Kruskal–Wallis test; ***P ≤ 0.001, *P ≤ 0.05, only significant differences are shown. (Scale bars, 100 nm.)

The localization of TSP-4 in MCGs that accumulate at the IS suggests that it might be released in association with SMAPs. To enhance GzmB imaging following SMAP release we used CTLs transfected with the GzmB-mCherry-encoding construct. The correct localization of GzmB-mCherry was confirmed by costaining with anti-GzmB antibodies (*SI Appendix*, Fig. S7*A*). Additionally, the colocalization of GzmB-mCherry with TSP-4 and TSP-1 at the IS formed on activating PSLBs recapitulated the one observed using anti-GzmB antibodies (*SI Appendix*, Fig. S7 *B* and C). For these experiments a GFPSpark-tagged TSP-4 construct was used (*SI Appendix*, Fig. S2). Next, CTLs were cotransfected with the TSP-4-GFPSpark and GzmB-mCherry constructs and plated on activating PSLBs to induce SMAP release, after which cells were flushed out by gentle washing. This leaves the synaptic output, that includes the SMAPs, on the PSLBs (13). Total Internal Reflection Fluorescence (TIRF)-based imaging showed a significant colocalization of TSP-4 and GzmB in particles of size compatible with SMAPs (Fig. 5*A*). Similar results were obtained when CTLs were cotransfected with TSP-1-GFPSpark and GzmB-mCherry (Fig. 5*B*). When CTLs were cotransfected with the constructs encoding TSP-4-mCherry and TSP-1-GFPSpark, TSP-4, and TSP-1 were found to colocalize in individual particles, of size compatible with SMAPs, released on the activating PSLBs (Fig. 5*C*).

To better define the identity of these particles as SMAPs and visualize the respective localization of TSP-4 and TSP-1 therein, we carried out a superresolution analysis of the synaptic output released by CTLs on activating PSLBs by direct STochastic Optical Reconstruction Microscopy (dSTORM). CTLs were cotransfected with the TSP-4-mCherry and TSP-1-GFPSpark constructs. The particles left on the PSLBs following CTL removal (8.8 particles/IS, Fig. 5 *D* and *E*) were stained with anti-GFP-AF488 and anti-RFP-AF555 for optimal blinking and WGA-AF647 to identify the SMAPs, and imaged by dSTORM (Fig. 5 *D* and *F* and *SI Appendix*, Figs. S8 and S9). As previously described for TSP-1 and WGA (13), TSP-4 displays ring staining consistent with its contributing to the glycoprotein shell of SMAPs (Fig. 5*F*). The size of SMAPs released at the IS (Fig. 5*G*) matched the size of MCG-associated SMAPs analyzed by CLEM (Fig. 2*E*). A diffraction limited single particle colocalization analysis, performed to increase the number of observations, showed a prevalence of TSP1^+^TSP4^+^WGA^+^ particles as compared to TSP1^+^WGA^+^ or TSP4^+^WGA^+^ ones (Fig. 5*H*), consistent with the CLEM and STED analysis of SMAPs within MCGs. Among the SMAPs single positive for either TSP released by cells coexpressing TSP-1-GFPSpark and TSP-4-mCherry, only a minor proportion were TSP1^+^WGA^+^ (Fig. 5*H*). Together, the data show TSP-4 is part of the SMAP shell and that TSP-4 and TSP-1 are coassembled and coreleased in individual SMAPs.

### TSP-4 Is Required for Full CTL- and SMAP-Mediated Killing.

While the specific role of SMAPs in CTL-mediated cytotoxicity is as yet not fully understood, TSP-1 deficiency in CTLs has been associated to impaired direct killing, although the contribution of SMAP-associated TSP-1 to this process has not been addressed (13). To assess the impact of TSP-4 deficiency on the killing ability of CTLs, CTLs transfected with TSP-4-targeting siRNAs or control scrambled siRNAs were mixed with SAg-pulsed target cells and cytotoxicity was monitored for 4 h by fluorimetry using the calcein release-based assay (26) (*SI Appendix*, Fig. S10*A*). TSP-4 knockdown (KD) CTLs showed an impaired ability to kill target cells (MEC1), similar to TSP-1 KD CTLs (*SI Appendix*, Fig. S10*A*). Similar results were obtained when killing was assessed by flow cytometry at different effector:target ratios (*SI Appendix*, Fig. S10*B*). Importantly, killing was restored when TSP-4 and TSP-1 KD CTLs were transfected with TSP-4-mCherry and TSP-1-mCherry, respectively (*SI Appendix*, Fig. S10*C*). Hence both TSP-1 and TSP-4 are required for full CTL-mediated killing.

**Fig. 6.**
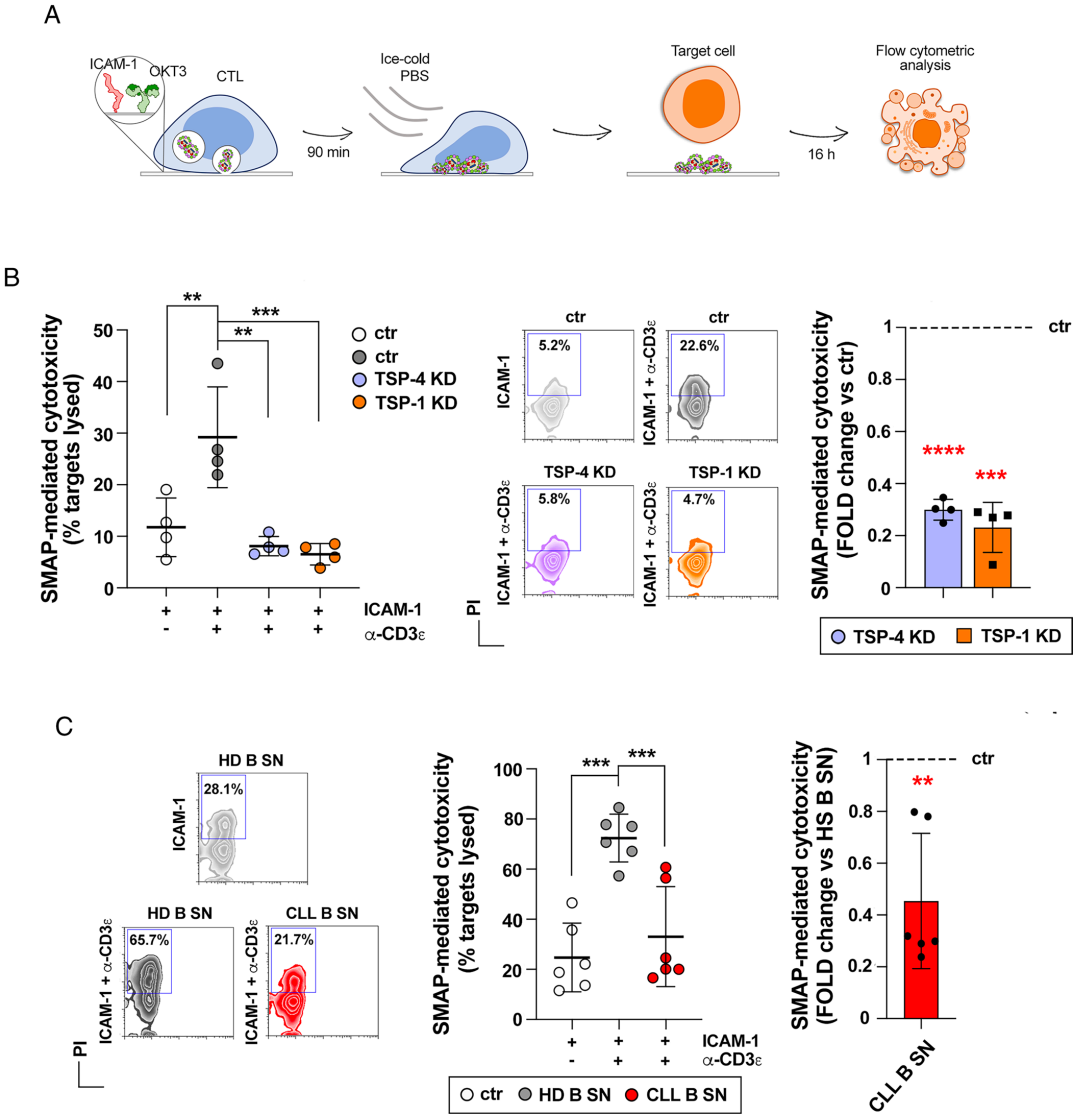
SMAP-mediated latent killing is dependent upon TSP-1 and TSP-4 and is decreased by soluble factors released from CLL. (*A*) Schematic diagram of the SMAP-mediated killing assay. (*B*) Flow cytometric analysis (mean ± SD) of cytotoxicity mediated by the synaptic output-including SMAPs-of control (ctr), TSP-4 KD, and TSP-1 KD CTLs plated on immobilized ICAM-1 or ICAM-1 + anti-CD3ε mAb. Quantification (mean ± SD) of target cell lysis (%) (*Left*), representative flow cytometry dot plots (*Middle*), and SMAP-mediated cytotoxicity expressed as fold change in KD samples vs. ctr (*Right*). N_donors_ = 4, one-way ANOVA test (*Left*) and one-sample *t* test (*Right*); ****P ≤ 0.0001, ***P ≤ 0.001, **P ≤ 0.01, only significant differences are shown. (*C*) Flow cytometric analysis (mean ± SD) of cytotoxicity mediated by the synaptic output of CTLs, generated in the presence of media conditioned by either healthy B cells (HD B SN) or B cells purified from CLL patients (CLL B SN) and plated on immobilized ICAM-1 or ICAM-1 + anti-CD3ε mAb surfaces. Quantification (mean ± SD) of target cell lysis (%) (*Left*), representative flow cytometry dot plots (*Middle*), and SMAP-mediated cytotoxicity expressed as fold change (*Right*) in samples treated with CLL B SN vs. samples treated with HD B SN. N_donors_ = 3 CD8^+^ samples from healthy donors treated with either 6 HD B SN or 6 CLL B SN, one-way ANOVA test (*Left*) and one-sample *t* test (*Right*); ***P ≤ 0.001, **P ≤ 0.01, only significant differences are shown.

To elucidate whether this function of TSP-4 is mediated by SMAPs, SMAPs released by control siRNA and TSP-4 siRNA–treated CTLs were recovered on glass surfaces coated with ICAM-1 and activating anti-CD3 antibodies. Glass surfaces coated with nonactivating ICAM-1 were used as negative control. MEC1 cells were then plated on the SMAPs captured on activating surfaces and incubated for 16 h (the earliest time at which significant killing was observed; legend *SI Appendix*, Fig. S10*E*), then recovered, stained with propidium iodide and analyzed by flow cytometry (Fig. 6*A*). The results show that SMAP-mediated cytotoxicity was impaired when SMAPs were released by TSP-4 siRNA–treated CTLs (Fig. 6*B*). This result was confirmed for SMAPs released by CTLs depleted of TSP-4 by CRISPR/Cas9 gene editing (*SI Appendix*, Fig. S10 *D* and E). The analysis was extended to SMAPs released by TSP-1 siRNA–treated CTLs. As shown in Fig. 6*B*, TSP-1 deficiency impaired SMAP-mediated killing. The data provide evidence that both TSP-4 and TSP-1 are required for latent killing by SMAPs.

### Impaired Cytotoxicity of CLL-Conditioned CTLs Is Associated with Impaired SMAP-Mediated Killing.

We have previously reported that leukemic cells from CLL patients shape the lymphoid microenvironment to promote their survival while suppressing anticancer immunity through both contact-dependent and contact-independent interactions (27, 28). In particular, we showed that CLL cells enhance their surface expression of the immunosuppressive ligand PD-L1 (29) and induce the upregulation of the inhibitory receptor PD-1 on CTLs (28), leading to impaired IS formation and CTL-mediated killing. We hypothesized that CLL cells could also directly suppress CTL function by tuning down the expression of the cytotoxic LG effectors. To test this hypothesis CD8^+^ T cells purified from healthy donors were differentiated to CTLs in the presence of culture supernatants from leukemic cells from CLL patients, using culture supernatants from healthy primary B cells as control (*SI Appendix*, Fig. S11*A*). As reported (28), CTLs generated in leukemic cell-conditioned media show an impaired ability to kill CFSE-stained target cells following activation in the presence of SAgs as assessed by flow cytometry, gating on CFSE-positive (CFSE^+^) cells and using propidium iodide to identify dead cells (*SI Appendix*, Figs. S11 *B* and C and S12 for gating strategy). RT-qPCR analysis of relative expression of shared SCG/MCG components (GzmA, GzmB, Prf, granulysin, serglycin) and of specific MCG/SMAP components (TSP-1, TSP-4) showed a reduction in the expression of GzmA, GzmB, serglycin, and TSP-4 (*SI Appendix*, Fig. S11*D*). Notably, TSP-1 mRNA levels were not affected under these conditions (*SI Appendix*, Fig. S11*D*). The results indicate that CLL cells disable CTLs not only through the PD-1-dependent impairment of IS assembly and function, but also by interfering with LG biogenesis.

The reduction of TSP-4 expression as well as of lytic effectors in CLL cell-conditioned CTLs suggests that the killing ability of SMAPs might be impaired. To test this, conditioned CTLs were plated on activating surfaces to capture the SMAPs. Killing by the SMAPs released by CLL cell-conditioned CTLs was reduced as compared to SMAPs derived from healthy B cell–conditioned CTLs, as assessed by plating MEC1 cells on the captured SMAPs followed by flow cytometric analysis of recovered cells stained with propidium iodide (Fig. 6*C*). Note that in this system there are no PD-1 ligands present. The results suggest that the SMAP biogenesis program of CTL is targeted by CLL cells for protection from CTL-mediated killing and further supports the relevance of TSP-4 and the cytotoxic function of SMAPs.

## Discussion

SMAPs have been identified as important element contributing to CTL and NK cell cytotoxicity (13, 16). However, how SMAPs are assembled, how they are released, and how they are taken up by target cells remains to be elucidated. The requirement for TSP-1 for full CTL-mediated killing (13) suggests that components of the glycoprotein shell participate in SMAP function. Among the SMAP glycoproteins identified by mass spectrometry, only TSP-1 and Galectin-1 have been investigated to date, however the latter appears dispensable for the cytotoxic activity of CTLs (13). Here, we have investigated the expression and function of TSP-4, which is among the glycoproteins picked up by the mass spectrometry analysis of material released by CTLs onto the PSLB during TCR mediated triggering (13), and that is related to, but also structurally distinct from TSP-1. Our evidence suggests that TSP-1 and TSP-4 are nonredundant components of SMAPs. We show that expression of TSP-4 and TSP-1 is regulated in a reciprocal manner during CD8^+^ T cell activation and differentiation to CTLs. TSP-4 colocalizes with TSP-1 in a population of LGs that corresponds to the MCGs and that accumulates at the IS, where TSP-4 is coreleased with TSP-1 in association with biologically active SMAPs. In cotransfection experiments TSP-4 reaches LGs faster than TSP-1, and endogenous TSP-4 enhances accumulation of TSP-1-GFPSpark in GzmB^+^ LGs. We show that TSP-4 as well as TSP-1 are required for the killing activity of SMAPs and may both play a role in CTL-mediated cytotoxicity. Additionally, we provide evidence that CLL cells produce soluble factors that reduce TSP-4 expression and compromise SMAP-mediated latent killing activity.

Expression of TSPs has been documented in a variety of cell types and tissues both in physiological and pathological conditions. While largely ubiquitous, expression of individual TSPs is spatiotemporally regulated, likely reflecting the requirement for these proteins in specific cellular processes (30–33). Here, we provide evidence that TSP-4 is expressed in CD8^+^ T cells throughout their differentiation to cytotoxic effectors. Interestingly, TSP-1 and TSP-4 show an opposite expression pattern during CD8^+^ T cell differentiation to CTLs, with a progressive increase in TSP-4 paralleled by a concomitant drop in TSP-1. The basal expression of TSP-1 may be associated with the effector memory T cell subset which was shown to have abundant TSP-1 positive SMAPs (13). Beyond their involvement in SMAP biogenesis and function, it remains to be elucidated whether TSP-1 and TSP-4 play distinct roles at other stages of CTL differentiation or in other effector functions. Alternatively, TSP-4 could take over the function of TSP-1 as expression of the latter declines. It is noteworthy that a similar opposite coregulation of TSP-1 and TSP-4 expression has emerged from the analysis of gene expression databases in brain and breast cancer (34–39). Our findings show that this can also occur in physiological conditions, with potential relevance to other cell types.

Our data show that TSP-1 and TSP-4 are preassembled in GzmB^+^LAMP-1^+^ vesicles in CTLs prior to IS formation. While the vesicular trafficking pathway that regulates their transport to these LGs remains to be characterized, only a proportion of GzmB^+^ vesicles are also TSP-1^+^ or TSP-4^+^, consistent with the existence of two classes of LGs, the SCGs and MCGs, that differ in morphology and protein composition, including the presence of TSP-1 (2) and TSP-4 (this report). This suggests a bifurcation of the GzmB trafficking pathway, with one branch leading to SCGs and another intersecting with TSP-1/4 trafficking at MCGs. Interestingly, our observation that TSP-4^+^TSP-1^+^ and TSP-4^+^ MCGs are more prevalent than TSP-1^+^ MCGs, highlighted by the CLEM and STED analysis and reflected at the SMAP level by the dSTORM analysis, suggested the hypothesis that TSP-1 may be incorporated into SMAPs mainly after TSP-4. Our finding, based on the RUSH experiments, that TSP-4 localizes indeed to LGs prior to TSP-1 and that, additionally, TSP-4 siRNA-mediated silencing reduces LG localization of TSP-1, indicates a diversification within the TSP-1/4 trafficking pathway and supports a tight, nonredundant interplay of the two TSPs in the stepwise process of SMAP biogenesis. It is noteworthy that the colocalization of both TSP-4 and TSP-1 with GzmB increases after activation, as observed in the PSLB experiments, suggesting a maturation step involving vesicle fusion triggered by CTL recognition of its cognate target leading to the release of fully armed SMAPs. Vesicle trafficking and fusion events during LG maturation and exocytosis have been reported previously for Rab11^+^Rab27^+^ endosomes and SCGs (40, 41), suggesting that similar mechanisms might apply to MCGs. We cannot however exclude that the results of the colocalization analyses might be overestimated due to LG clustering under activating conditions which may affect discrimination of individual vesicles.

Using activating PSLBs, we found that the preformed TSP-1^+^/TSP-4^+^ LGs accumulate at the IS and that TSP-1 and TSP-4 are coreleased as SMAPs endowed of latent killing activity. Owing to the lack of antibodies suitable for high-quality imaging, these observations were carried out using fluorescently tagged TSP-1 and TSP-4. While this provides information on the relative localization of the two TSPs within CTLs prior and following stimulation, they do not answer the issue of the relative SMAP content of endogenous TSP-4 vs. TSP-1. The mass spectrometry analysis captured a substantially larger abundance of TSP-1 vs. TSP-4 peptides (13), suggesting that, despite the downregulation in TSP-1 expression, the residual amounts are sufficiently high to play a role in SMAPs and, accordingly, TSP-1 KO was shown to impair the killing ability of CTLs (13) and of SMAPs (this report). Here, we show that TSP-4 deficiency leads to defects both in CTL- and SMAP- mediated killing in the absence of any exogenous TSP expression, indicating that, despite the fact that we do not have sufficient information as to the relative amounts of endogenous TSP-4 vs. TSP-1 at the single-cell and single-SMAP level, both TSPs participate in SMAP biogenesis and function. *Thbs1*^−/−^ and *Thbs4*^−/−^ mice may help unravel this issue. Both mice are viable but display a number of developmental and functional defects, from major lung abnormalities in *Thbs1*^−/−^ mice (42) to neuronal and cardiac muscle defects in *Thbs4*^−/−^ mice (43, 44). Immune functions of these mice have not been examined to date, although an increase in white blood cell numbers, including lymphocytes, has been observed in *Thbs1*^−/−^ mice (42). While both TSP-1 and TSP-4 are required for the killing activity of SMAPs, they might participate in CTL-mediated killing by affecting also other key features of the killing process, one of these being CTL adhesion to target cells, that TSPs might affect through binding to integrins and other adhesion molecules (45).

Tumor cells have evolved a variety of strategies to escape elimination by CTLs, including preventing their recruitment, enhancing the generation and function of Tregs by promoting the expression of inhibitory checkpoints, and inhibiting the assembly of functional ISs, as exemplified by CLL (46–48). We have previously reported that CLL cells have the ability to rewire cellular components of the lymphoid niche, which is their primary tumor microenvironment, to enhance their homing to and persistence therein, where they are exposed to survival factors and protected from apoptosis-inducing drugs. These include stromal cells, which upregulate their production of homing chemokines (27), and CTLs themselves, which upregulate PD-1 expression (28). Here, we identified an additional immunosuppressive strategy whereby CLL cells disable CTLs by downregulating the expression of key components of their cytotoxic machinery. Interestingly, these include not only cytotoxic molecules shared by SCGs and MCGs, but also TSP-4. Taken together with the killing defect of SMAPs released by TSP-4 deficient CTLs, the fact that SMAPs released by CTLs exposed to the culture supernatants of CLL cells have an impaired killing ability further highlights the importance of TSP-4 in SMAP function, although further work is required to determine whether SMAPs play an important role in killing of CLL cells.

Our study has a number of limitations. While we have analyzed endogenous TSP-1 and TSP-4 mRNA and immunoblotting of proteins, we found that the commercial antibodies to TSP-1 and TSP-4, while confirming the specificity of their fluorescent counterparts, were unsuitable for high-quality immunofluorescence studies on fixed cells. Thus, all of single-cell analysis and subcellular localization studies were performed with fluorescent protein or epitope-tagged TSPs in primary CD8^+^ T cells. This may lead to an underestimation of the number of TSP^+^ MCGs or SMAPs as not all CTLs express the recombinant constructs. This could account for the lower number of SMAPs/IS compared to earlier reports (13, 16), although a reduction in the efficiency of SMAP biogenesis by transfected vs. native CTLs could also contribute to the difference. Additionally, while TSP-4 expression increases and TSP-1 expression decreases following activation, we expressed TSP-1-GFPSpark and TSP-4-mCherry at similar level, which may also impact the frequency of double and single-positive LGs and released SMAPs in our analysis. However, the droplet digital PCR analysis showed that *THBS1* and *THBS4* are expressed at comparable levels in differentiated CTLs, supporting the results obtained on CTLs expressing the recombinant fluorescently tagged proteins. Finally, TSP-1/4 KD CTLs show a defect in early (4 h) CTL-mediated cytotoxicity, while SMAPs released by TSP-1/4 KD CTLs only show defects at later times (16 h) when SMAP-mediated killing could be detected. This could suggest that TSP-1/4 secreted through the canonical pathway to become incorporated into the extracellular matrix (17) may exploit their adhesive properties to favor CTL interaction with target cells and thus facilitate killing in an SMAP-independent fashion, in addition to their role in latent SMAP-mediated cytotoxicity. Alternatively, the difference in timing may be due to technical limitations of how SMAPs are delivered in the different assays. Active CTL scanning for MEC1 targets may result in rapid SMAP delivery allowing assessment of TSP-1/4 dependence in 4 h, whereas encounters of nonsubstrate adherent MEC1 targets with substrate-attached SMAPs may take longer, leading to a need for 16 h to assess TSP-1/4 dependence.

In conclusion, our data identify TSP-4 as a nonredundant participant in SMAP biogenesis and function. This not only advances our knowledge of the mechanisms underpinning CTL-mediated killing but also potentially paves the way to SMAP-targeted, therapeutic strategies through engineering T cell TSPs to remove the molecular determinants responsible for nonselective cell adhesion (innate recognition), while endowing them with tumor antigen specificity.

## Materials and Methods

### Reagents.

Antibodies are documented in *SI Appendix*, Table S1 and oligonucleotides are listed in *SI Appendix*, Table S2.

### Cell Culture and Gene Expression.

All studies were performed with human peripheral blood CD8^+^ T cells purified using the RosetteSep negative selection kit from deidentified blood products and activated in vitro with anti-CD3 + anti-CD28 coated Dynabeads in RPMI-1640 with 10% fetal bovine serum and 50 U/mL IL-2 for the time and treatments as indicated. Expression of TSPs and other constructs was carried out by plasmid electroporation using an Amaxa Nucleofector II system (Lonza). siRNA were introduced using the Amaxa Nucleofector II system on day 3 of culture and cells used 24 h later. Cas9 protein complexes with gRNA were introduced by electroporation on day 3 and cell used 2 to 3 d later.

### Microscopy.

TIRF and spinning disc confocal microscopy were carried out on Nikon ECLIPSE Ti2-E microscope equipped with a Yokogawa CSU-W1-SoRA; STED was carried out on Abberior instruments GmbH QUADScan; SIM on ultrathin sections on a Zeiss ELYRA PS.1 microscope; and dSTORM on an ONI Nanoimager microscope. The multiparameter SIM images were correlated with grayscale images acquired with a Tecnai 12 Biotwin electron microscope.

### RUSH Assay.

The RUSH system was established for TSP-1 and TSP-4 through addition of a SBP tag and coexpression with streptavidin tagged with an endoplasmic retention signal [KDEL or the ER retention signal of the ER-resident Ii isoform (25)] to retain the TSPs in the endoplasmic reticulum until addition of biotin (#4698, Merck) at a final concentration of 40 µM (49). Analysis of TSP localization was then performed at different times after addition of biotin.

### CLL Supernatants.

Healthy control or CLL B cells were incubated at 3.3 × 10^6^/mL for 48 h to prepare conditioned media that was then used at 50% to prepare CTLs from human peripheral blood CD8^+^ T cells as above.

### Statistical Testing.

Tests were performed using GraphPad software. Tests for normality of distribution were performed. Normally distributed data were compared using parametric tests. Nonnormally distributed data were compared using nonparametric tests. *P* values were corrected for multiple testing as required.

More details are included in *SI Appendix*, *Further Methods* and Tables S1 and S2.

## Supplementary Material

Appendix 01 (PDF)

## Data Availability

Datafiles have been deposited in an Open Science Framework project 10.17605/OSF.IO/4GZSM (50). This will include protocols and analysis macros. Materials not available from Addgene or other commercial sources will be made available on request. All other data are included in the manuscript and/or *SI Appendix*.
