# Supplementary material for: Activation-induced thrombospondin-4 works with thrombospondin-1 to build cytotoxic supramolecular attack particles
Source: Proc Natl Acad Sci U S A. 2025 Feb 4;122(6):e2413866122. doi: 10.1073/pnas.2413866122 (PMC11831147; doi:10.1073/pnas.2413866122)
Supplement: Supplementary file 1 — Appendix 01 (PDF) [file pnas.2413866122.sapp.pdf]

Further methods

Ethics

Either buffy coats (Siena University Hospital) or leukocyte reduction system chambers, a by-product of platelet collection from healthy blood donors (Institute of Clinical Hemostaseology and Transfusion Medicine, Saarland University Medical Center; University of Oxford Biobank, Memorial Blood Center, St. Paul, Minnesota, USA) were obtained from the local blood banks. All healthy donors and CLL patients provided voluntary informed written consent to use their blood for research purposes. All procedures were performed according to the Declaration of Helsinki guidelines. Research with human PBMC and CLL-derived samples has been approved by the local ethic committee of Siena University (20759; Prof. Baldari), Saarland University (98/15; Dr. Krause), Oxford University (REC 11/H0711/7; Prof. Dustin), University of Minnesota (No approval required) and Padua University (23229; Prof. Trentin).

CLL patients

Peripheral blood samples were collected from 16 previously untreated CLL patients. Diagnosis of CLL was made according to international workshop on CLL (iwCLL) 2008 criteria (1). The immunophenotypic analysis of lymphocytes obtained from peripheral blood of CLL patients was performed as reported (2). B cells from 16 buffy coats, used as healthy population controls, were purified by negative selection using RosetteSep B-cell enrichment Cocktail (StemCell Technologies, Vancouver, Canada) followed by density gradient centrifugation on Lympholite (Cedarlane Laboratories, The Netherlands), as reported (2).

Plasmids and antibodies

Expression constructs for human TSP-4 tagged with fluorescent tags were generated by

insertion of their respective coding sequences into a pMax backbone. GFPSpark and mCherry cDNAs were amplified using the primers listed in Table S2, and pCMV-GzmB-mCherry (3) and pCMV3-TSP1-GFPSpark (4) as templates, then inserted into the pMax via restriction digestion (Thermo Fisher Scientific) and ligation by T4 DNA ligase (Promega Corporation). TSP-4 cDNA was amplified from pRP[Exp]-mCherry/Neo-CAG>hTHBS4[NM_003248.6] (ID VB900137- 1200xfw, VectorBuilder) removing the terminal STOP codon by using primers listed in Table S2 and inserted into the pMax-GFPSpark and pMax-mCherry vectors using KpnI and EcoRI restriction sites. The pMax-GFPSpark and pMax-mCherry vectors (empty vectors) were used as negative controls. The TSP-1-GFPSpark coding sequence was subcloned from pCMV3-TSP-1-GFPSpark (4) to a pMax vector using KpnI and XbaI restriction sites. The constructs were confirmed by sequencing. TSP-1 cDNA was amplified from pCMV3-TSP1-GFPSpark (4) by using primers listed in Table S2 and inserted into the pMax-mCherry vector using KpnI and HindIII restriction sites. The human GzmB-mCherry construct (3) was kindly provided by Prof. Jens Rettig.

To generate pMax-TSP-1-linker-3xFLAG, the TSP-1 gene sequence was amplified from the background vector pMax-TSP-1-GFPSpark using the primers listed in Table S2 to add KpnI and XbaI sites at the 5’ and 3’ ends. The linker and the 3xFLAG were generated through the annealed oligo cloning method, using the primers listed in Table S2. The oligos were mixed in equimolar amounts, heated to 95°C for 5 min, and gradually cooled to room temperature. The annealed linker was digested with XbaI and NheI, and 3xFLAG with NheI and XhoI. Subsequently, the background plasmid vector pMax was digested with KpnI and XhoI restriction enzymes. The digested products were then ligated into the pMax-digested vector, generating the pMax-TSP-1-linker-3xFLAG vector. Cloning of pMax-TSP-4-linker-3xHA plasmid was achieved by first digesting the pMax- THBS4-mCherry vector with EcoRI and XhoI restriction sites to remove the mCherry tag. Then, the linker was generated

through the annealed oligo cloning method, using the primers listed in Table S2. The annealed linker was digested with EcoRI and XbaI. The HA tag was digested from the pMax-3xFLAG-mFwe 2-3xHA vector using XbaI and XhoI restriction enzymes. The digested linker and HA tag were then ligated with the pMAX-TSP-4-digested vector to obtain the final clone. The final vectors were confirmed by sequencing the plasmids using the respective forward and reverse primers, a process conducted by Microsynth Seqlab.

To clone the reporter of interest (i.e. TSP-4 and TSP-1) into the RUSH plasmids, the sequence encoding human TSP-1 fused to a streptavidin binding peptide (SBP) was synthetized in vitro (ID VB210412-1326qnm, VectorBuilder) and AscI and SbfI sites were added at the 5' and 3' ends. Both insert and the Ii-Str_TNF-SBP-EGFP (#65280, Addgene) vector were digested using AscI and SbfI (New England Biolabs) and ligated by T4 DNA ligase (Promega Corporation) after removing the TNF coding sequence. Human TSP-4 cDNA was amplified from pRP[Exp]-mCherry/Neo-CAG>hTHBS4[NM_003248.6] (ID VB900137-1200xfw, VectorBuilder) by using the primers listed in Table S2 and inserted into the Str- KDEL_TNF-SBP-mCherry (#65279, Addgene) (5) via restriction digestion with AscI and EcoRI (Thermo Fisher Scientific) and ligation by T4 DNA ligase (Promega Corporation) after removing TNF coding sequence. The constructs were confirmed by sequencing.

Primary commercial antibodies used in this work and their application are listed in Table S1. The bacterial superantigens (SAgs) staphylococcal enterotoxins A (SEA), B (SEB) and E (SEE) were purchased from Toxin Technology Inc. Bovine serum albumin (BSA) heat shock fraction (pH 7), poly-L-lysine, propidium iodide and saponin were obtained from Merck, Triton X-100 and BSA fraction V (pH 7) from PanReac Applichem.

RNA purification, RT-qPCR and droplet digital PCR

Relative target levels were quantified using RT-qPCR. RNA was extracted from $0.3x10^5$-

$5x10^6$ $CD8^+$ T cells using the RNeasy Plus Mini Kit and quantified using a QIAxpert spectrophotometer (Qiagen). 500 ng of RNA for each sample were reverse transcribed to first-strand cDNA iScript cDNA Synthesis Kit, and analyzed by quantitative PCR (qPCR) using the SSo Fast™ Eva Green® Super Mix (Bio-Rad Laboratories) and the CFX96 Real-Time PCR Detection System (Bio-Rad Laboratories). After an initial denaturation at 95°C for 3 min, samples were subjected to 42 cycles of denaturation at 95°C for 10 sec and primer annealing plus extension at 60°C for 30 sec, followed by 10 sec at 95°C and a denaturation gradient from 65°C to 95°C with 0.5°C increment every 5 sec to generate melt curves. Results were processed and analyzed using the CFX Manager Version 1.5 software (Bio-Rad). The relative abundance of *GLNY, GZMA, GZMB, PRF, SRGN, THBS1* and *THBS4* transcripts, and of *THBS1* and *THBS4* in knock-down samples, was determined on duplicate samples using the ΔΔCt method (6) and normalized to 18S ribosomal RNA or to HPRT1 RNA. Specific primers used to amplify cDNA fragments corresponding to human transcripts are listed in Table S2.

Absolute quantification of *THBS4* and *THBS1* mRNA copies were quantified using the droplet digital PCR (Bio-Rad). 5 μl of cDNA were added to a 20 μl PCR reaction mixture containing 5 μl of ddPCR Multiplex Supermix, 1.2 μl of PrimePCR™ ddPCR™ Gene Expression Probe Assay (FAM) *THBS4* (ID: dHsaCPE5034816; Bio-Rad Laboratories) and 0.7 μl of PrimePCR™ ddPCR™ Gene Expression Probe Assay (HEX) *THBS1* (ID: dHsaCPE5037121; Bio-Rad Laboratories) and DNAse/RNA-free distilled water (Invitrogen™). Droplets were generated loading the mixture into a plastic cartridge with 70 μl of Droplet Generation oil (Bio-Rad Laboratories). Cartridges were then placed into a QX100™ Droplet Generator (Bio-Rad Laboratories), then generated droplets were transferred to a 96-well PCR plate and PCR amplification was carried out on a C1000 Touch Thermal Cycler (Bio-Rad Laboratories) according to the manufacturer's thermal protocol. After PCR the plate was loaded on the QX100™ Droplet Reader (Bio-Rad

Laboratories) and each sample automatically read. Positive and negative fluorescence data from the sample droplets were acquired and analyzed by the QuantaSoft software 2.2 (Bio-Rad Laboratories) using Poisson distribution. The total number of *THBS4* and *THBS1* copies/μl of reaction was provided by the software, then multiplied for the reaction volume and divided for the volume of cDNA added to the reaction mixture to express data as number of *THBS4* and *THBS1* copies/μl of cDNA that in our specific case corresponds to 25 ng of RNA.

Cell lysis, immunoblots and immunoprecipitations

Primary CD8$^+$ T cells were washed twice with ice-cold PBS 1X and lysed in 20 mM Tris-HCl pH 8, 150 mM NaCl, 1% Triton X-100 in the presence of Protease Inhibitor Cocktail Set III (Calbiochem®) and 0.2 mg sodium orthovanadate for 5 min on ice. The homogenates were centrifuged at 16,100 x g for 20 min at 4°C and the soluble fractions were recovered. Protein concentration in post-nuclear supernatants was determined using the Quantum Protein Bicinchoninic Acid (BCA) Assay (Euroclone) and the iMark™ microplate reader (Bio-Rad Laboratories) to measure the absorbance at 570 nm. 10 µg of protein extract (post-nuclear supernatant) were denatured in reduced 1X Bolt™ LDS Sample Buffer (Invitrogen™) for 5 min at 100°C and resolved on precast polyacrylamide gradient gels Bolt™ 4 to 12%, Bis-Tris, 1.0 mm, Mini Protein Gels (Invitrogen™) in 1X Bolt™ MES SDS Running Buffer with a constant voltage of 150V. Separated proteins were transferred to nitrocellulose membranes (Amersham Protran®) in 20 mM Tris, 0.2 M glycine, 20% (v/v) ethanol for 75 min setting a constant current of 250 mA. Membranes were blocked in 4% (w/v) non-fat dry milk in PBS 1X (140 mM NaCl, 2.7 mM KCl, 10 mM phosphate buffer pH 7.4) + 0.02% (v/v) Tween-20 (PBS-T), incubated with primary abs (Table S1) followed by horseradish peroxidase-labeled goat anti-mouse or anti-rabbit secondary IgG (heavy and light chains) (Jackson ImmunoResearch Inc) (Table S1), and revealed using the

chemiluminescent detection kit SuperSignal™ West Pico PLUS Chemilumiscent Substrate (Thermo Fisher Scientific). Chemiluminescence signals were acquired by using Alliance Q9-ATOM imaging system (UVITEC, Cambridge, UK) and NineAlliance x64 software (UVITEC). The densitometric analysis of protein bands was carried out using ImageJ (National Institutes of Health, USA).

For coimmunoprecipitation experiments, $7x10^6$ CTLs expressing TSP-4-mCherry in combination with either GFPSpark alone or TSP-1-GFPSpark were lysed in 20 mM Tris-HCl pH 8, 150 mM NaCl, 1% Triton X-100 in the presence of Protease Inhibitor Cocktail Set III (Calbiochem®) and 0.2 mg sodium orthovanadate for 5 min on ice. Postnuclear supernatants were incubated with 2.2 μg Protein A Sepharose CL-4B (PAS; #GEH17078001, GE Helathacare) for 1 h at 4°C. PAS were pelleted by centrifugation (PAS controls) and supernatants were transferred to new tubes plus 0.2 μg of anti-GFP antibodies for 3 h at 4°C. PAS controls and PAS-antibody complexes were then washed four times with IP lysis buffer. The immunoprecipitates were diluted in 2x Bolt™ Sample Reducing Buffer (#B009, Life Technologies), boiled for 5 min and resolved by SDS-PAGE. A fraction (10 μg) of the lysates used of co-IPs was run on the same gel to identify the migration of specific immunoreactive bands.

Flow cytometry

Flow cytometry analysis of surface CD62L and CD45RA was carried by incubating 0.2-$0.15x10^6$ $CD8^+$ T cells in the dark for 30 min on ice with APC-labeled anti-hCD62L and FITC-labeled anti-hCD45RA (Table S1). Viable cells were gated based on size and granularity. Sample acquisition was performed with a Guava® easyCyte™ Flow Cytometer (Merck Millipore) using the appropriate lasers and emission filters, and data were analyzed using FlowJo 6.1.1 software (TreeStar Inc.). Staining was performed in two independent experiments with cells from different donors. To determine the cells single positive for each

marker and both double negative and double positive, the density plot was split into four quadrants and the relative abundance (%) of CD45RA and CD62L within viable cells identified $CD45RA^+/CD62L^+$ naïve cells, $CD45RA^-/CD62L^+$ central memory cells, $CD45RA^-/CD62L^-$ effector memory cells and $CD45RA^+/CD62L^-$ terminal effector memory re-expressing CD45RA (TEMRA) cells in the samples was then quantified.

For flow cytometry analysis of CTLs transiently transfected with the constructs encoding TSP-1-GFPSpark, TSP-4GFPSpark and TSP-4-mCherry (alone or in combination), 24 h after transfection $0.2x10^6$ cells were fixed with Cyto-Fast Fix/Perm Solution (#750000133, BioLegend) in the dark for 15 min at room temperature and labelled with anti-GFP and anti-RFP primary antibodies in Cyto-Fast Perm/Wash solution 1X (#750000135, BioLegend) for 30 min on ice followed by washing with Cyto-Fast Perm/Wash solution 1X and labelling with AF488-anti-mouse or AF647-anti-rabbit antibodies in Cyto-Fast Perm/Wash solution 1C for 30 min on ice in the dark. Untransfected cells and cells with only secondary antibodies were used as negative controls. Transfection efficiency was calculated as the percentage of cells showing above-background levels of fluorescence. In double transfectants transfection efficiency was comparable for the two constructs in all experiments.

Flow cytometry was perfomed using a Guava® easyCyte™ Flow Cytometer and guavaSoft InCyte 2.7 software (Merck Millipore) using the appropriate lasers and emission filters. Data were analyzed using guavaSoft InCyte 2.7 (Merck Millipore) and FlowJo 6.1.1 (Tree Star Inc.).

Cell culture and transfection of CTLs for confocal imaging

Primary human $CD8^+$ T lymphocytes were isolated from buffy coats of anonymous healthy donors and purified (>95% purity) by negative selection using the RosetteSep™ Human $CD8^+$ T Cell Enrichment Cocktail (STEMCELL Technologies). Buffy coats were collected

at the Siena University Hospital after obtaining written informed consent from each donor and processed following standard ethical procedures outlined in the Declaration of Helsinki. Freshly purified $CD8^+$ T cells (day 0) were activated by adding Dynabeads™ Human T-Activator CD3/CD28 (Gibco™) at a cell/bead ratio of 1:0.5 for 48-64 h and expanded in complete R10 medium [RPMI-1640 medium containing 20 mM HEPES and 2.05 mM L-glutamine (#7388, Merck) supplemented with 10% iron-enriched bovine calf serum (BCS; GE Healthcare HyClone), 120 µg/ml penicillin, 1% MEM Non-Essential Amino Acids (MEM NEAA; Gibco™) and 100 U/ml of recombinant human rhIL-2 (Miltenyi) for further 3-5 d (days 5 and 7 after isolation) to generate CTLs as previously reported (7).

CTLs were transiently transfected with the constructs pMax-TSP-1-GFPSpark, pMax-TSP-4-GFPSpark, pMax-TSP-4-mCherry, pMax-GzmB-mCherry constructs, and the GFPSpark and mCherry control vectors (DNA/cell ratio = 1.5 µg/10^6 cells in single transfections and 1.2 µg/10^6 cells in cotransfections) using the Human T Cell Nucleofector Kit (Amaxa Biosystem) and the Amaxa Nucleofector II system (Lonza), Program T-023. Cells were cultured with 35 U/ml of rhIL-2 in complete R10 medium at a cell density of $1x10^6$ cells/ml and analyzed 24 h after transfection. 8 h after transfection, cells were incubated with 5 µg WGA-AF647 (#W32466, Invitrogen™) for 16 hours at 37°C in a humidified 5% CO2 incubator.

Spinning Disk Confocal Microscopy imaging (SDCM)

3D confocal microscopy imaging was carried out on fixed samples at 200 nm steps using a Nikon ECLIPSE Ti2-E microscope equipped with a Yokogawa CSU-W1-SoRA spinning disk confocal unit and a Photometrics Prime BSI (Nikon), and NIS-Elements AR 5.42.02 64-bit software. A 60X/1.49 NA oil immersion objective were used for image acquisition. For SDCM imaging, cells were plated on 10-well diagnostic microscope slides (Epredia) coated with 0.1% (w/v) poly-L-lysine (Merck) in H2O, fixed 4% PFA/PBS (v/v) for 15 min at room

temperature. After fixation, cells were washed in PBS 1X (v/v), permeabilized and stained with primary abs (Table S1) in 1% (w/v) BSA, 0.1 (v/v) % Triton X-100 or saponin in PBS 1X over-night at 4°C. Samples were washed with PBS 1X (v/v) and incubated with fluorescent secondary abs (Table S1) for 45 min at room temperature. Samples were washed again with PBS 1X (v/v), mounted in 90% glycerol/PBS (v/v) and inspected by SDCM.

NIS-Elements software (Nikon) was used to process the acquired data sets. This software performs denoising and deconvolution using the Richardson-Lucy algorithm and 10 iteractions to obtain images with enhanced resolution and improved signal-to-noise ratio. Postprocessing and analysis of fluorescence images was performed with Fiji (National Institutes of Health). Colocalization analyses on median optical sections and z-stacks was performed using JACop plug-in (8) to calculate the Manders' overlap coefficient (MOC). MOC varies from 0 to 1, with 0 standing for non-overlapping images and 1 for 100% colocalization between two images. Specifically, M1 is defined as the ratio of the "summed intensities of pixels from the green image for which the intensity in the red channel is above zero" to the "total intensity in the green channel" and M2 is defined conversely for red (9).

PSLB preparation

PSLB were prepared as previously described (4, 10). Small unilamellar vesicles composed of 0.4 mM solution of lipids in PBS with 75 mol% 1,2-dioleoyl-sn-glycero-3-phosphocholine supplemented (DOPC) and 25 mol% 1,2-dioleoyl-sn-glycero-3-[(N-(5-amino-1-carboxypentyl) iminodiacetic acid) succinyl] (DOGS-NTA) (Avanti Polar Lipids Inc) were prepared by extrusion using the Avanti Miniextruder with a 100 nm filter. Protein concentrations required to achieve desired densities on bilayers were calculated from calibration curves that were constructed from flow cytometric measurements of fluorescent proteins attached on bilayers formed on glass beads, compared with reference molecules

of equivalent soluble fluorophores beads (Bangs Laboratories).

Sticky 6-channel slides (sticky-Slide VI 0.4, Ibidi) were glued to cleanroom cleaned coverslips 0.170x0.005 mm thickness (SCHOTT MINIFAB Diagnostics – NEXTERION® Glass). A 1:1 mixture of DOGS-NTA lipids and DOPC stocks were loaded in each channel and incubated for 20 min to generate mobile PSLBs. Channels were washed twice with 0.1% (w/v) BSA in HEPES Buffered Saline 1X pH 7.2 (0.2 mM HEPES, 1.37 mM NaCl, 50 nM KCl, 7 nM Na_2HPO_4, 60 nM D-glucose, 1 mM $CaCl_2$, 2 mM MgCl2) and blocked with 2% BSA/HBS (w/v) supplemented with 100 µM $NiSO_4$ to generate Ni-chelating lipids that anchor His-tagged proteins. The channels were washed again twice with 0.1% BSA/HBS before adding 200 molecules/µm^2 AF405-labeled ICAM-1 alone (steady-state conditions) or in combination with 30 molecules/µm^2 unlabeled anti-CD3ε (UCHT1) Fab' (activating conditions). Excess proteins were removed by washing twice with 0.1% BSA/HBS.

IS formation on activating surfaces

CTLs were plated onto either nonactivating (ICAM-1) and activating (ICAM1 + anti- CD3ε Fab') surfaces for 30 min or nonactivating [poly-L-lysine, PLL] and activating [30 µg anti-CD3ε OKT3 (#317302, BioLegend)] for 15 min at 37°C and 5% CO2, then fixed with 4% paraformaldehyde (PFA) in PBS 1X (v/v) for 15 min at room temperature. After fixation, samples that had been activated on SLBs were washed twice with 0.1% BSA/HBS, blocked and permeabilized with 1% (w/v) BSA, 0.1 (v/v) % Triton X-100 in PBS 1X for 1 h at room temperature and then stained with primary abs (Table S1) overnight at 4°C. Samples were washed twice with 0.1% BSA/HBS and incubated with fluorescent secondary abs (Table S1) for 45 min at room temperature. Samples were washed again twice with 0.1% BSA/HBS and analyzed by SDCM.

Total Internal Reflection Fluorescence (TIRF) imaging of released SMAPs

CTLs were plated onto nonactivating (ICAM-1) and activating (ICAM1 + anti-CD3ε Fab') PSLBs and incubated for 90 min at 37°C and 5% CO_2, then flushed out by washing three times with ice-cold PBS. SMAPs released by CTLs and remained attached to PSLBs were fixed with 4% PBA/PBS (v/v) for 15 min at room temperature and analyzed by TIRF microscopy using a Nikon ECLIPSE Ti2-E microscope equipped with a Yokogawa CSU-W1-SoRA spinning disk confocal unit and a Photometrics Prime BSI (Nikon). A 60X/1.49 NA oil immersion objective was used for image acquisition.

Cell culture and transfection of human CTLs for CLEM and STED microscopy

Human PBLs were obtained from healthy donors as previously described in (11). Naïve $CD8^+$ T cells were isolated using the Dynabeads™ untouched human CD8 T-cell isolation kit (Invitrogen), stimulated with Dynabeads™ Human T-Activator CD3/CD28 (Gibco™) and cultured for 5 days in AIMV medium supplemented with 10% fetal calf serum (FCS) and 100 U/ml of rhIL-2 (Gibco). $8x10^6$ CTLs were transfected with 6 µg of plasmid DNA of each construct with P3 Primary Cell 4D- Nucleofector X Kit (Lonza). For the CLEM experiments the cells were transfected with TSP-1-GFPSpark and TSP-4-mCherry, while for the STED microscopy experiments the CTLs were transfected with TSP-1-Flag and TSP-4-HA (12). Cells were washed 6 h after plasmid transfection and replated in fresh medium. 16 h posttransfection, cells were either directly immunostained for STED microscopy or incubated with WGA-AF647 (1:1000) for 2 hours prior the CLEM experiment. After two wash steps in ice cold RPMI-1640 medium, cells were collected in AIM V medium with 30% FCS and high pressure frozen.

Postembedding Correlative Light Electron Microscopy (CLEM)

Workflow is shown in Fig.S4A. CTLs were seeded on sapphire discs coated with poly-L-ornithine (0.1 mg/ml) and anti-CD3 UCHT1 (30 µg/ml, BioLegend #300402) in flat specimen

carriers (Leica). After incubation at RT (20°C ± 2°C) for 15 min, samples were vitrified in a high-pressure freezing system (Leica EM PACT2). Samples were further processed in a freeze-substitution apparatus (AFS2; Leica) as described in (2). Briefly, all samples were transferred to the precooled (−130°C) freeze-substitution chamber of the AFS2. The temperature was increased from −130 to −90°C over 2 h. Freeze substitution was performed from −90 to −70°C over 20 h in anhydrous acetone and from −70 to −60°C over 24 h with 0.3% (w/v) uranyl acetate in anhydrous acetone. At -60 °C samples were infiltrated with increasing concentrations (30, 60, and 100% for 1 h each) of Lowicryl (3:1 K11M/HM20 mixture; Electron Microscopy Sciences). After 5 h of 100% Lowicryl infiltration, samples were UV polymerized at −60°C for 24 h and for additional 15 h at a linear temperature increase to 5°C. Samples were stored in the dark at 4°C until further processing. After removal of the carriers and the sapphire discs ultrathin sections (100 nm) were cut using an UC7 (Leica) and collected on carbon-coated 200 mesh copper grids (Plano). 1-d after sectioning the grids were stained with DAPI for 3 min (1/1000), washed and sealed between two coverslips for high-resolution SIM imaging. The same grids previously analyzed with SIM were stained with uranyl acetate and lead citrate and recorded with the Tecnai 12 Biotwin electron microscope. Only CTLs with well-preserved membranes, cell organelles and nuclei were analyzed and used for correlation. For correlation, the DAPI 405 nm image showing the labeled nucleus of the cell was used to find the optimal overlay with the electron microscope images. The final alignment defines the position of the fluorescent signals within the cell of interest. The Images were overlaid in Corel DRAW 2021.

Structured Illumination Microscopy (SIM)

For high-resolution SIM (ELYRA PS.1; Carl Zeiss Microscopy) of the ultrathin sections, images were acquired by using the 63X Plan-Apochromat (NA 1.4) objective with excitation light of 405-, 488-, 561- and 642-nm wavelengths to visualize DAPI, TSP-1-GFPspark, TSP-

4-mCherry and WGA-AF647, respectively. The DAPI image was recorded to identify both the nucleus of the CTL and the image plane. In a z-stack analysis 3-10 images were recorded with a step size of 100 nm to scan the cells of interest. ZEN 2012 software (Zeiss) was used for data acquisition and image processing to achieve higher resolution.

Immunostaining for STED microscopy

Cells were seeded on coverslips, which were coated with poly-L-ornithine (0.1 mg/ml) and mouse anti-CD3 UCHT1 (30 µg/ml, BioLegend # 300402), for 10 min at RT (20°C ± 2°C) to allow IS formation. They were then fixed for 20 min with 4% PFA at room temperature and autofluorescence was quenched by 10 min incubation with 50 mM Glycine. The cells were permeabilized and blocked with 0.1% Triton X-100 and 5% normal goat serum (NGS) in PBS for 10 min. To prevent unspecific binding of secondary antibody on the mouse anti-CD3 antibodies, the samples were blocked for 1 h with 5% NGS in PBS, washed and treated for an additional hour with Fab' anti-mouse (1:50 in PBS with 5% NGS, Rockland # 8111102). Then the cells were washed and incubated with the specified primary abs (Table S1). After two washing steps, the samples were incubated with secondary abs for 45 min at room temperature (20 ± 2 °C). After several washing steps the samples were mounted with antifade mounting medium (Abberior #MM-2013-2X15ML).

STimulated Emission Depletion (STED) microscopy

Imaging was performed with a four-colour STED QUADScan (Abberior instruments GmbH) using 485 nm/0.85 mW, 561 nm/2 mW, and 640 nm/12 mW excitation pulsed lasers, and a 775 nm / 1.25 W STED laser. The pinhole size was set to 90 µm (1.13 arbitrary unit) and the probes were visualized with a 100X/NA 1.4 objective (UPLSAPO100XO, Olympus). The following acquisition protocol was applied using Imspector software (Abberior instruments GmbH). First, a single confocal section was recorded at 488 nm, 561 nm, and

640 nm to identify cells containing GzmB, TSP-1- Flag, and TSP-4-HA staining. Then, one section at the IS was acquired in confocal mode at 488 nm for GzmB and in 2DSTED mode at 561 nm and 647 nm to visualize the TSP-1 and TSP-4 staining. The laser power was 20%, 5% and 10% at 488, 561 and 640 nm, respectively. The STED laser emitted 50% of the maximal power of 1200 mW (corresponding to 73–83 mW in the focus, with a repetition rate of 40 MHz). The pixel size was 20×20 nm, pixel dwell time was 5 µs, and the line accumulation 4. Finally, a stack of 1 µm depth was recorded of the same IS in confocal mode for GzmB and in 3DSTED mode for TSP-1-Flag and TSP-4-HA. The laser powers were 10, 3 and 7 % respectively. The STED laser emitted at 60% (corresponding to 87–99 mW in the focus, with a repetition rate of 40 MHz, set at 50% of 3D capacity). The voxel size was 20×20×50 nm, pixel dwell time was 5 µs, and the line accumulation 2. Laser power had to be slightly adjusted depending on the combination of secondary antibodies that were used.

STED image analysis

Single plane images acquired at the IS, in which GzmB labelling was acquired in confocal mode and TSP-1 and TSP-4 labelling were acquired in 2D STED mode, were segmented using Cellpose 2.0 (14). Images were background subtracted using ImageJ (15) then the „Fire“ look up table was applied and the image was save as RGB. We used the “cyto” model as starting point to train Cellpose 2.0 on our images over 4 to 5 rounds. Training was performed separately for each channel. After training the images were segmented automatically. In few cases manual adjustments had to be made. The segmented images were saved and subsequently used in an in house written Matlab (MathWorks) program classifying the overlapping objects according to their staining. Objects were deemed to be the same within the different channels if at least 10% of their surface overlapped.

Tracking TSP-4 and TSP-1 transport to LGs by the Retention Using the Selective Hooks (RUSH) system

3x10^{6} of 3-d activated CD8^{+} T cells were transfected using the Human T Cell Nucleofector Kit (Amaxa Biosystem) and the Amaxa Nucleofector II system (Lonza), Program T-023 with 1.5/10^{6}. Cells were cultured with 50 U/ml of rhIL-2 in RPMI-1640 medium (#8758, Merck) supplemented with 7.5% iron-enriched BCS (GE Healthcare HyClone) for 24 h and then used for a time-course of release by adding biotin (#4698, Merck) at a final concentration of 40 µM as previously described (16). A sample of transfected cells incubated 60 min in medium without biotin was used as negative control. Cells were fixed with 4% PFA in PBS 1X (v/v) for 15 min at room temperature, permeabilized and stained with primary abs (Table S2) in 1% (w/v) BSA, 0.1 (v/v) % saponin in PBS 1X overnight at 4°C, then incubated with fluorescent secondary abs (Table S1) for 45 min at room temperate.

Cell culture and transfection of human CTLs for dSTORM microscopy

Isolation of peripheral blood CD8^{+} T cells from the buffy coat of healthy donors using the RosetteSep™ Human CD8^{+} T cell Enrichment Cocktail, as per manufacturers protocol, and subsequent centrifugation at 1,200 xg from 20 minutes with no brake at RT in a Ficoll gradient. CD8^{+} resting population are washed in R10 (RPMI-1640 medium (#7388, Merck) with 2 mM L-Glutamine, supplemented with 10 % FBS, 2 mg/ml penicillin, with 100 U/ml of rhIL-2) to remove residual Ficoll prior to culturing in R10 at 1x10^{6} cell/mL. CD8^{+} resting T cells were activated using Dynabeads™ Human T-activator CD3/CD28 in a 1:1 ratio with cells. Cells were seeded into a flat bottom 24 well plate at 1x10^{6} cell/ml including Dynabeads and incubated at 37 °C in a humidified 5% CO_2 incubator. Beads were removed 48 h after activation and then used for nucleofection.

Nucleofection was performed using the Amaxa T Cell nucleofector kit. 2x10^{6} cells per condition were centrifuged at 200 xg for 10 minutes at RT, resuspended in 100µl of RT

Human T Cell Nucleofector Solution then combined with 1.5 µg of total DNA (TSP-1-GFPSpark and TSP-4-mCherry). The cell/DNA suspension was placed in the nucleofection cuvette and run using the T-023 Nucleofector program. Pre-equilibrated R10 culture medium was added to the cuvette then transferred into a 24 well plate to a final volume of 1ml (R10 with 50u/ml rIL2)/well/sample. Nucleofected cells were rested for 24 h, checked for double construct expression, then deposited on the PLSB as previously described.

2D Single Molecule Localization Microscopy (SMLM) and direct Stochastic Optical Reconstruction Microscopy (dSTORM)

The Oxford NanoImager (ONI) microscope was used to generate 2D SMLM images using dSTORM. A 100X objective lens/1.4 NA in oil-immersion was used. 405 nm, 488 nm, 561 nm and 640 nm lasers were used with 2 channels (with dichroic mirror split at 640 nm). All calibrations, controls and images were performed at 27°C with a supercritical TIRF angle of 54°.

After flushing out CTLs following 1.5 h of SMAP deposition on the PSLB (ICAM1 + anti-anti-CD3ε UCHT1 Fab'), channels were first fixed for 30 min at 4°C with 4% PFA + 0.25% Glutaraldehyde, washed 6 x with MilliQ, then incubated with anti-RFP-AF555 and anti-GFP-AF488 overnight to optimize sample blinking during image acquisition. Ibidi channels were washed 6 x with MilliQ and then incubated with WGA-AF647 for 8 hours, then washed again. Samples were imaged in dSTORM buffer (320 µl 1x PBS, 40 µl 50% glucose, 40 µl 2-Mercaptoethylamine HCl (2-MEA, 4 µl glucose-oxidase). First, the 640 nm laser was used at 75% laser power to excite WGA-AF647, then the 561 nm laser was used at 80% laser power to excite AF555, followed by the 488 nm laser to excite AF488. The 405 nm laser was used to promote fluorophore blinking. 3,000 frames were acquired per fluorophore. An exposure of 30 ms was used. Calibration channel mapping performed using TetraSpec Microspheres of 100 nm were used to align all three channels and generate the final three-

coloured-dSTORM image using CODI software.

The Oxford NanoImager (ONI) analysis platform, CODI, was used to analyse 3 colour dSTORM images. Localization, acquisition, and mapping files were uploaded for each image taken. Drift correction was applied using the DME algorithm. Frames were filtered first according to acquisition program settings then to exclude the initial excitation peaks for each laser. The steady state of localizations per frame was included as true blinking localizations. Filtering parameters around the sigma peak were then applied to include a sigma range of ~50-250 nm (Gaussian fit to localize single molecules). Photon counts with a value about 300-500 were included to ensure good signal-to-noise. Localization precision filtering was then applied to include localization with precision less than or equal to 20 nm. SMAP radius and number of localizations was determined using the contained clustering algorithm using the radius and locs functions applied to the region of interest (the area of SMAP deposition at the c-SMAC). Micrographs of SMAPs were depicted with a display sigma of 5 nm using fixed representation. Files were downloaded from CODI in TIFF and PNG formats then cropped and converted to PNG micrographs for figures.

RNA interference, CRIPSR/Cas9 genome editing and rescue

For RNAi-mediated TSP-1 and TSP-4 silencing $4x10^6$ 3-d activated $CD8^+$ T cells were transfected using the Human T Cell Nucleofector Kit (Amaxa Biosystem) and the Amaxa Nucleofector II system (Lonza), Program T-023 with 150 pmol/10^6 cells of human TSP-4 and TSP-1-specific siRNAs (#s14108 and #s14100, Invitrogen™), and control scrambled siRNAs (#4390846, Invitrogen™). Cells were tested for knockdown efficiency by qPCR and used for assays 24 h posttransfection. To investigate the association of TSP-1 with LGs in TSP-4 KD CTLs, we cotransfected $3x10^6$ 3-day activated $CD8^+$T cells with 150 pmol/10^6 cells of human TSP-4-specific siRNAs (#s14100, Thermo Fisher Scientific) and 1.5 μg/10^6 of the pMax-TSP-1-GFPSpark construct. Cells were tested for TSP-1-GFPSpark

expression by flow cytometry and for knock-down efficiency by qPCR 24 h posttransfection, and they were fixed and labelled for immunofluorescence microscopy.

For CRISPR-Cas9 genome editing 2x10^6 2-d activated $CD8^+$ T cells were transfected using ribonucleoprotein complexes prepared by mixing 10 µg of Alt-R® S.p. Cas9 Nuclease V3 protein (Integrated DNA Technologies, #1081059) and 6 µg of target-specific guide RNAs (gRNA) for TSP-1 and TSP-4 (Table S2), which were designed using the web tool CRISPOR.org (17), transcribed *in vitro* using HiScribe™ T7 High Yield RNA Synthesis Kit (#E2040S, NEB) and purified with RNA Clean & Concentrator™ (#R1017, Zymo Research). Cells were grown in complete R10 medium supplemented with 500 U/ml of rhIL-2 for 3 d, then tested for knockout efficiency by immunoblotting.

For rescue experiments, 4x10^6 3-d activated $CD8^+$ T cells were transfected using the Human T Cell Nucleofector Kit (Amaxa Biosystem) and the Amaxa Nucleofector II system (Lonza), Program T-023 with 150 pmol/10^6 cells of human TSP-4 and TSP-1-specific siRNAs (#s14108 and #s14100, Thermo Fisher Scientific) in combination with either the TSP-4-mCherry or TSP-1-mCherry constructs (DNA/cell ratio = 1.5 µg/10^6 cells in single transfections) in the respective KD and the mCherry-expressing control constructs. Cell transfected with control scrambled siRNAs (#4390846) (Invitrogen™) were cotransfected with the mCherry-expressing control constructs. Cells were analyzed by qPCR and used for assays 24 h posttransfection.

Analysis of CTL-mediated and SMAP-mediated cytotoxicity

B cell lines used as target cell lines (i.e. Raji and MEC1) were grown at 37°C and 5% CO_2 in RMPI-1640 medium (Merck) supplemented with 7.5% iron-enriched BCS (GE Healthcare HyClone).

For the flow cytometry analysis of cell-mediated cytotoxicity (20), Raji B cells or MEC1 B cells, used as targets, were stained with 1.5 µM carboxyfluorescein diacetate succinimidyl

ester (CFSE; #C34554; Invitrogen™) for 8 min at room temperature in PBS and then pulsed with a mixture of the Staphylococcal superantigens (SAgs) (SEA, SEB, and SEE, 2 μg/ml of each) for 1 h in serum-free AIM V™ medium (#12055-091; Gibco™). Unpulsed CFSE-stained target cells were used as negative control. $30x10^4$ target cells were mixed up with 7-d conditioned CTLs at different E:T ratios in 60 μl of AIM V™ medium and incubated at 37°C and 5% CO_2 for 4 h. For killing assays using transfected and/or KD/KO CTLs, where viability was impaired and yield reduced, E:T ratio(s) leading to efficient killing without reaching saturation (Fig.S1D) were used. Thereafter, samples were diluted to 200 μl with cold PBS and acquired using a Guava Easy-Cyte flow cytometer (Merck) after adding 20 μg/ml propidium iodide (PI; #537059, Merck) to stain dead cells. Target cells alone and target cells lysed with 1% Triton X-100 (Calbiochem) were used for instrument set up and for gating $CFSE^+PI^+$ cells (Fig.S12 for gating strategy). Cytotoxicity (% target cell lysis) was calculated as follows: ($CFSE^+PI^+$ cells − $CFSE^+PI^+$ cells in control sample) × 100 / (100 − $CFSE^+PI^+$ cells in control sample).

For real-time calcein release-based analysis of cell-mediated cytotoxicity (18, 19), MEC1 B cells were stained with 500 nM calcein-AM (#C1430, Invitrogen™) in serum-free AIM V™ medium (#12055-091; Gibco™) with 10 mM HEPES at room temperature for 15 min, then washed twice and pulsed with a mixture of staphylococcal superantigens A, B, and E (SAgs, 2 μg/ml of each) for 1 h. $30x10^4$ MEC1 cells were plated in a 96-well black plates with clear bottom (BD Falcon) and CTLs were added at a effector-target (E:T) ratio of 3:1. Cytotoxicity were measured at 37°C and 5% CO_2 every 10 min for 4 h (an incubation time that led to efficient killing without reaching saturation; Fig.S1E) using a Synergy HTX multi-mode plate reader (BioTek) at 485 nm excitation and 528 nm emission wavelengths in the bottom reader mode. Cytotoxicity (% target cell lysis) was calculated as follows: (F_{live} − γ x F_{exp}) / (F_{live} − F_{lyse}) x 100, where F_{live} is the fluorescence of targets alone, F_{exp} are target+CTL samples and F_{lyse} is the maximal target lysis in the presence of 1% Triton X-100 (Calbiochem). The γ

value was measured at time zero: $\gamma = F_{live}(0)/F_{exp}(0)$. All the experiments were performed in duplicate and averaged to obtain a value per experiment.

For the flow cytometry analysis of SMAP-mediated cytotoxicity, sticky 6-channel slides (sticky-Slide VI 0.4, Ibidi) were glued to cleanroom cleaned coverslips 0.170x0.005 mm thickness (SCHOTT MINIFAB Diagnostics – NEXTERION® Glass) and coated with 10 µg/ml rhICAM-1/Fc Chimera (#720-IC-200, R&D), in presence (stimulated condition) or absence (unstimulated condition) of 5 µg/ml anti-human CD3ε (clone OKT3; #317302, BioLegend) in PBS at 37°C for 2h. Thereafter, 1-0.5-1x10^6 CTLs/well were plated onto immobilized ICAM-1 or ICAM-1 + anti-CD3ε mAb for 90 min at 37°C and 5% CO_2, then CTLs were flushed out for three times with ice-cold PBS and released SMAPs, which had been captured on coated glass surfaces, were incubated with 0.5-1x10^6 MEC1 cells/well for 16 h at 37°C and 5% CO_2. After an overnight incubation, MEC1 cells were recovered, stained with 20 µg/ml PI (Merck) and dead cells analyzed by flow cytometry. Unstained cells were used as negative control. Target cell death was calculated as the percentage of PI^+ cells gated based on the negative control.

Conditioning of $CD8^+$ T cells by CLL cell supernatants

Culture supernatants were prepared by growing 5x10^7 B cells, purified from peripheral blood of either healthy donors or CLL patients by negative selection using RosetteSep™ Human B-cell enrichment Cocktail (#15064; STEMCELL Technologies), in 15 ml RPMI-1640 medium (#8758, Merck) supplemented with 7.5% BCS (GE Healthcare HyClone) for 48 h. Samples were centrifuged and supernatants were stored at -80°C.

Freshly isolated $CD8^+$ cells were activated with Dynabeads™ Human T-Activator CD3/CD28 (Gibco™) at a cell/bead ratio of 2:1 for 48 h in conditioned media obtained by combining R10 medium and culture supernatants derived from either leukemic or healthy B cells (1:1 ratio). Fresh R10 medium was used as control. After 48 h, beads were removed

and activated CD8$^+$ T cells were expanded in conditioned media supplemented with fresh rhIL-2 for further 3 to 5 d.

Statistics

Statistical analyses were performed using GraphPad Prism software version 6.07 (GraphPah). N represents the number of biological replicates (donors) and n is the number of technical replicates (cells, granules and SMAPs analyzed); both are specified in each figure legend. At least three independent experiments, except for data shown in Fig. 2, 3, 4B-C, 5D-F (N=2), were performed for each assay. Normality of data distribution was tested using Anderson-Darling test, D'Agostino-Pearson omnibus normality test, Shapiro-Wilk normality test and Kolmogorov-Smirnov normality test with Dallal-Wilkinson-Lilliefor P value and normality assumption was accepted only when all datasets follow a normal distribution. Unpaired student's t-test (unpaired) and one-way/two-way ANOVA tests followed by post-hoc Tukey correction test and multiple comparisons were used to determine the significance between two or multiple groups of datasets with normal distribution. Mann-Whitney *U* test or Kruskal-Wallis nonparametric test followed by post-hoc Dunn's correction test were used to determine the significance between two or multiple groups of non-normally distributed datasets. One-sample t-test was used to compare the mean of one or more samples to a known standard mean of 1 or 100 assigned to control samples. Statistical analyses were performed using the Prism software (GraphPad Software). Statistical significance was defined as: ns $P > 0.05$; * $P \leq 0.05$; ** $P \leq 0.01$; *** $P \leq 0.001$; **** $P \leq 0.0001$.

References

1. M. Hallek *et al.*, iwCLL guidelines for diagnosis, indications for treatment, response assessment, and supportive management of CLL. *Blood* 131, 2745-2760 (2018).

2. L. Patrussi, N. Capitani, C. T. Baldari, Interleukin (IL)-9 Supports the Tumor-Promoting Environment of Chronic Lymphocytic Leukemia. *Cancers (Basel)* 13, 6301 (2021).
3. M. Ming, C. Schirra, U. Becherer, D. R. Stevens, J. Rettig, Behavior and Properties of Mature Lytic Granules at the Immunological Synapse of Human Cytotoxic T Lymphocytes. *PLoS One* 10, e0135994 (2015).
4. S. Balint *et al.*, Supramolecular attack particles are autonomous killing entities released from cytotoxic T cells. *Science* 368, 897-901 (2020).
5. G. Boncompain *et al.*, Synchronization of secretory protein traffic in populations of cells. *Nat Methods* 9, 493-498 (2012).
6. M. W. Pfaffl, A new mathematical model for relative quantification in real-time RT-PCR. *Nucleic Acids Res* 29, e45 (2001).
7. A. Onnis *et al.*, SARS-CoV-2 Spike protein suppresses CTL-mediated killing by inhibiting immune synapse assembly. *J Exp Med* 220, e20220906 (2023).
8. S. Bolte, F. P. Cordelieres, A guided tour into subcellular colocalization analysis in light microscopy. *J Microsc* 224, 213-232 (2006).
9. E. M. M. Manders, F. J. Verbeek, J. A. Aten, Measurement of co-localization of objects in dual-colour confocal images. *J Microsc* 169, 375-382 (1993).
10. P. Demetriou *et al.*, A dynamic CD2-rich compartment at the outer edge of the immunological synapse boosts and integrates signals. *Nat Immunol* 21, 1232-1243 (2020).
11. E. C. Schwarz *et al.*, Calcium dependence of T cell proliferation following focal stimulation. *Eur J Immunol* 37, 2723-2733 (2007).
12. N. Alawar, C. Schirra, M. Hohmann, U. Becherer, A solution for highly efficient electroporation of primary cytotoxic T lymphocytes. *BMC Biotechnol* 24, 16 (2024).
13. H. F. Chang *et al.*, Identification of distinct cytotoxic granules as the origin of

supramolecular attack particles in T lymphocytes. *Nat Commun* 13, 1029 (2022).

14. M. Pachitariu, C. Stringer, Cellpose 2.0: how to train your own model. *Nat Methods* 19, 1634-1641 (2022).
15. J. Schindelin *et al.*, Fiji: an open-source platform for biological-image analysis. *Nat Methods* 9, 676-682 (2012).
16. N. Capitani, C. Cassioli, K. Ravichandran, C. T. Baldari, Exploiting the RUSH System to Study Lytic Granule Biogenesis in Cytotoxic T Lymphocytes. *Methods Mol Biol* 2654, 421-436 (2023).
17. J. P. Concordet, M. Haeussler, CRISPOR: intuitive guide selection for CRISPR/Cas9 genome editing experiments and screens. *Nucleic Acids Res* 46, W242-W245 (2018).
18. C. Kummerow *et al.*, A simple, economic, time-resolved killing assay. *Eur J Immunol* 44, 1870-1872 (2014).
19. H. F. Chang *et al.*, Cytotoxic granule endocytosis depends on the Flower protein. *J Cell Biol* 217, 667-683 (2018).
20. A. Kabanova *et al.*, Human Cytotoxic T Lymphocytes Form Dysfunctional Immune Synapses with B Cells Characterized by Non-Polarized Lytic Granule Release. *Cell Rep* 15, 9-18 (2016).

Supplementary figure legends

Figure S1. Generation of effector CD8$^+$ T cells. (**A**) Timeline of the protocol used for *in vitro* generation of cytotoxic CD8$^+$ T cells acquiring cytolytic activity (day 5 and 7 after purification) from primary CD8$^+$ T lymphocytes (day 0) purified from peripheral blood of healthy donors. (**B**) *Left*, Representative dot plot showing the percentage (%) of naïve (T_{naive}, CD62L$^+$/CD45RA$^+$), central memory (T_{CM}, CD62L$^+$/CD45RA$^-$), effector memory (T_{EM}, CD62L$^-$/CD45RA$^-$) and terminal effector memory re-expressing CD45RA (T_{EMRA}, CD62L$^-$/CD45RA$^+$) in freshly isolated CD8$^+$ T cells (day 0) and differentiated 5-d and 7-d CTLs generated as depicted in (**A**). *Right*, The graph (mean±SD) shows the percentage of naïve, central memory, effector memory and late effector memory re-expressing CD45RA detected in 5-d CTL cultures of 5 representative donors. (**C**) RT-qPCR of *GZMB* and *PRF* in freshly isolated CD8$^+$ T cells (day 0) and 5-d and 7-d CTLs. The graphs (mean±SD, ctr value = 1) show the abundance of the analysed transcripts, which was determined using the DDCt method and normalized to 18S ribosomal RNA. $N_{donors} = 6$, one-sample t-test; * $P \leq 0.05$, only significant differences are shown. Each dot represents one donor. (**D**) Flow cytometry analysis of cell-mediated cytotoxicity by differentiated CTLs that were mixed with SAg-pulsed CFSE-stained target B cells at different E:T ratios (i.e. 3:1, 6:1, 9:1). Cytotoxicity was assessed by using propidium iodide (PI) uptake by target cells. The graph shows the percentage (mean±SD) of CFSE$^+$/PI$^+$ cells. $N_{donors} = 2$, one-way ANOVA test; ** $P \leq 0.01$, * $P \leq 0.05$. (**E**) Fluorimetric analysis of cell-mediated cytotoxicity by differentiated CTLs using a calcein release-based assay. The curve shows the kinetics of target cell death by CTLs generated as depicted in A at the effector:target ratio of 3:1. $N_{donors} = 2$. (**F,G**) Validation of anti-TSP-4/1 antibodies for immunoblotting in KO CTLs. Immunoblot analysis of TSP-4 (**E**) and TSP-1 (**F**) in lysates of control and TSP-4 KO or TSP-1 KO CTLs gene-edited by CRISPR-Cas9 technology. Actin was used as loading control. The migration of molecular

mass markers is indicated (kDa).

Figure S2. Analysis of TSP-1-GFPSpark, TSP-4-mCherry and TSP-4-GFPSpark expression and localization in transfected CTLs. (**A**) Schemes of the expression constructs with 3’ fluorescent tags encoding full-length TSP-1 and TSP-4 fused to the N-terminus of GFPSpark (GFPS) and mCherry (mC), respectively. (**B**) Representative FACS profile of CTLs expressing mC alone or TSP-4-mC and stained with an anti-RFP antibody. (**C**) Representative immunoblots of lysates of CTLs expressing either mC alone or TSP-4-mC. Samples were probed with the anti-TSP-4 antibody. Actin was used as a loading control. The migration of molecular mass markers is indicated (kDa). (**D**) Confocal images (medial optical sections) of 4-d CTLs expressing either mC alone or TSP-4-mC. Dashed lines mark the cell outline. (Scale bar, 5 µm.) (**E**) Representative FACS profiles of CTLs expressing GFPS alone, TSP-1-GFPS (*Top*) or TSP-4-GFPS (*Bottom*) and stained with anti-GFP antibody. (**F**) Representative immunoblots of lysates of CTLs expressing either GFPS alone, TSP-1-GFPS (*Left*) or TSP-4-GFPS (*Right*). Samples were probed with anti-TSP-1 and anti-TSP-4 antibodies. Actin was used as a loading control. The migration of molecular mass markers is indicated (kDa). (**G**) Confocal images (medial optical sections) of CTLs expressing GFPS alone (*Top*), TSP-1-GFPS (*Middle*) or TSP-4-GFPS (*Bottom*). Dashed lines mark the cell outline. (Scale bar, 5 µm.) (**H,I**) Validation of anti-TSP-4/1 antibodies for immunofluorescence. (**H**) Confocal images (medial optical sections) of CTLs expressing either TSP-4-mC (*Top*) or TSP-1-GFPS (*Bottom*) and co-stained with anti-TSP-4 and anti-TSP-1 antibodies, respectively. Dashed lines mark the cell outline. (Scale bar, 5 µm.) (**I**) Quantifications (mean±SD) of the weighted colocalization using the Manders’ overlap coefficient between TSP-4-mC and anti-TSP-4 signals (*Left*) and between TSP-1-GFPS and TSP-1 signals (*Right*).

Figure S3. TSP-4 co-localizes with TSP-1 in LGs. (**A,B**) *Top*, Confocal images (medial optical sections) of CTLs expressing either mCherry-tagged TSP-4 (**A**) or GFPSpark-tagged full-length TSP-1 (**B**) co-stained with antibodies against GzmB, Prf, LAMP-1 or GM130. Dashed lines mark the cell outline. (Scale bar, 5 μm.) *Bottom*, Quantification (mean±SD) of the weighted colocalization using the Manders' overlap coefficient between TSP-4 (**A**) or TSP-1 (**B**) staining and the signals of each marker. $N_{donors} = 3$, $n_{cells} \geq 28$, Kruskal-Wallis test; **** $P \leq 0.0001$, only significant differences are shown. Each dot represents one cell. (**C**) *Left*, Confocal images (medial optical sections) of CTLs transiently co-transfected with constructs encoding TSP-4-mCherry and TSP-1-GFPSpark. Dashed lines mark the cell outline. (Scale bar, 5 μm.) *Middle*, Quantification (mean±SD) of the weighted colocalization using the Manders' overlap coefficient between TSP-4 and TSP-1 staining $N_{donors} = 3$, $n_{cells} \geq 28$, Kruskal-Wallis test; only significant differences are shown. Each dot represents one cell. *Right*, Quantification (%) of vesicles single or double positive for TSP-4 and TSP-1. (**D**) Immunoblot analysis of GFP-specific immunoprecipitates from lysates of CTLs expressing either GFPS- or TSP-1-GFPS and cotransfected with TSP-4-mCherry construct. A preclearing control (proteins that bound to Protein-A-Sepharose before addition of primary antibodies) is shown (neg ctr). Total cell lysates (lys) were included in each gel to identify the migration of recombinant TSP-4/1. The migration of molecular mass markers is indicated (kDa).

Figure S4. Human CTLs contain typical MCGs and SCG. (**A**) Workflow for the four-colour postembedding CLEM procedure**.** (**B**) Representative TEM images of 6 human CTLs without electroporation. Shown are TEM images of human CTLs (*Left*). The white rectangles mark the magnified area in the images on the right. White arrows indicate typical multicore granules (MCGs) characterized by multiple dense cores (SMAPs). The black arrow indicates a typical single core granule (SCG) (*Right* panel, *Lower* image). $N_{donors} = 3$, $n_{cells} = 21$.

Figure S5. TSP-1 and TSP-4-containing LGs concentrate at the IS. (**A**) Schematic representation of the IS formed between a CTL and PSLBs presenting laterally mobile ICAM-1 and anti-CD3ε UCHT1 Fab'. The architecture of a mature IS is characterized by an ICAM-1-enriched ring (blue) surrounding an inner secretory domain, where MCGs containing SMAPs and SCGs are focally released. (**B**) 3D view of a representative CTLs coexpressing TSP-4-mCherry (red) and TSP-1-GFPSpark (green), and interacting with either nonactivating [ICAM1-AF405] (*Top*) or activating [ICAM1-AF405 + anti-CD3ε UCHT1 Fab' (unlabeled)] ligands (*Bottom*) for 30 min. After fixation, cells were permeabilized and co-stained with anti-GzmB (magenta) antibodies. The formation of a mature IS is indicated by the presence of an ICAM-1 ring (blue). (Scale bar, 2 µm.) (**C**) Quantifications (mean±SD) of the 3D weighted colocalization on z-stacks using the Manders' overlap coefficient between each TSP signal and GzmB staining (Fig. S6 for individual donors). $N_{donors} = 3$, $n_{cells} \geq 6$ cells, Mann-Whitney test; *** $P < 0.001$, ** $P < 0.01$. Each dot represents one cell. (**D,E**) Quantifications (mean±SD) of the 3D weighted colocalization on z-stacks using the Manders' overlap coefficient between each TSP signal and WGA staining (**D**), and between TSP-4 and TSP-1 signals (**E**). $N_{donors} = 3$, $n_{cells} \geq 10$ cells, Mann-Whitney test; only significant differences are shown. Each dot represents one cell. (**F**) 3D view of a representative CTL coexpressing TSP-4-mCherry (red) and TSP-1-GFPSpark (green), preincubated with WGA-AF647 (magenta) and plated on either nonactivating [poly-L-lysine, PLL] (*Top*) or activating [anti-CD3ε OKT3 mAb (unlabeled)] (*Bottom*) for 15 min. (Scale bar, 2 µm.)

Figure S6. Colocalization analysis of TSP-4, TSP-1 with GzmB in unstimulated or activated CTLs. (**A,B**) 3D weighted colocalization using the Manders' overlap coefficient between TSP-4 and GzmB staining (**A**) and between TSP-1 and GzmB staining (**B**) in

individual donors. $N_{donors} = 3$, $n_{cells} \geq 6$ cells, Unpaired t-test; **** $P < 0.0001$, *** $P < 0.001$. Each graph shows the data set of a single donor, and each dot represents one cell.

Figure S7. GzmB-mCherry signal largely overlaps anti-GzmB staining and co-localizes with TSP-4 and TSP-1 at the IS. (**A**) *Left*, Confocal images (medial optical sections) of CTLs expressing GzmB-mCherry and co-stained with an anti-GzmB antibody. Dashed lines mark the cell outline. (Scale bar, 5 µm.) *Right*, Quantifications (mean±SD) of the weighted colocalization using the Manders' overlap coefficient between GzmB-mCherry signal with anti-GzmB staining. (**B,C**) 3D view of a representative CTLs expressing either TSP-4-GFPSpark (green) (**B**) or TSP-1-GFPSpark (green) (**C**) and interacting with activating [ICAM1-AF405 + anti-CD3ε UCHT1 Fab' (unlabelled)] ligands (*Right*) for 30 min, followed by staining with an anti-GzmB antibody (magenta). The formation of a mature IS is indicated by the presence of an ICAM-1 ring (blue). (Scale bar, 5 µm.)

Figure S8. **Quality control and analysis pipeline of 2D SMLM dSTORM acquisitions.** (**A**) Light program settings for 2D SMLM dSTORM imaging using the ONI. The Rainbow LUT is used to depict the sequence of localisation acquisitions from 0-3000 (647), 3001-6000 (555) and 6001-9000 (488), indicated as the frame index. (**B**) The image analysis pipeline following upload of raw files into the CODI analysis platform. (**C,E**) Frame index micrographs of triple and double positive particles demonstrating localisations of each colour were acquired during the appropriate laser excitation frame period (shown by the dotted lines). (**D,F**) The localization precision and sigma peak corresponding to the images of the particle in (**C,E**). The base of the localization precision peak at 20 nm indicates a resolution of 20 nm was achieved. The sharp, single Gaussian peak on the sigma plot indicates the image acquisition had good fluorophore blinking quality and therefore accurate molecule

localization.

Figure S9. 2D SMLM dSTORM of CTL-derived SMAPs at the IS. (**A**) Micrographs of particles deposited at the IS by CTLs on the PSLB with particles of interest denoted by grey boxes. (Scale bar, 2 µm.) (**B**) Table summarizing the analysis of each particle category based on average diameter, particle number and number of size outliers. (**C**) Additional representative micrographs of all three particle categories. SMAPs ware stained with WGA-AF647, TSP-4-mCherry was probed with anti-RFP-AF555 and TSP-1-GFPSpark was probed with anti-GFP-AF488. Scale bars are indicated in each panel row.

Figure S10. TSP-4 is required for CTL- and SMAP-mediated cytotoxicity. (**A**) Fluorimetric analysis of cytotoxicity mediated by control (ctr, TSP-4 and TSP-1 mRNA levels = 1), TSP-4 KD (TSP-4 mRNA = 0.57±0.19) and TSP-1 KD (TSP-1 mRNA 0.31±0.15) CTLs using the calcein release-based assay. Representative curves showing the kinetics of target cell death by CTLs at the effector:target ratio of 3:1 (*Left*). Quantification (mean±SD) of target cell lysis (%) after 4 h (*Middle*) and cell-mediated cytotoxicity expressed as fold change in KD samples versus ctr (*Right*). N_{donors} = 3, one-way ANOVA test; *** $P \leq 0.001$, **$P \leq 0.01$, * $P \leq 0.05$, only significant differences are shown. (**B**) Flow cytometric analysis of cell-mediated cytotoxicity by control (ctr, TSP-4 and TSP-1 mRNA levels = 1), TSP-4 KD (TSP-4 mRNA = 0.57±0.19) and TSP-1 KD (TSP-1 mRNA 0.31±0.15) CTLs mixed with SAg-pulsed CFSE-stained MEC1 B cells, used as targets, at different E:T ratios. Cytotoxicity was assessed by using propidium iodide (PI) uptake by target cells. The graph shows the percentage (mean±SD) of $CFSE^+/PI^+$ cells. N_{donors} = 5, two-way ANOVA test; **** $P \leq 0.0001$, ** $P \leq 0.01$, * $P \leq 0.05$. (**C**) Flow cytometric analysis of cell-mediated cytotoxicity by control (ctr, TSP-4 and TSP-1 mRNA levels = 1), TSP-4 KD (4-KD, TSP-4 mRNA = 0.56±0.33), TSP-1 KD (1-KD, TSP-1 mRNA = 0.41±0.24) CTLs and KD CTLs in which the expression

of TSP-4 (4-KD + TSP-4-mC, TSP-4 mRNA = 14.10±7.06) or TSP-1 (1-KD + TSP-1-mC, TSP-1 mRNA = 11.80±6.62) was restored by transfection with TSP-4-mCherry and TSP-1-mCherry constructs, respectively. CTLs were mixed with SAg-pulsed CFSE-stained MEC1 B cells, used as targets, at different E:T ratios. Cytotoxicity was assessed by using propidium iodide (PI) uptake by target cells. The graph shows the percentage (mean±SD) of $CFSE^+/PI^+$ cells. $N_{donors} \geq 4$, two-way ANOVA test; **** $P \leq 0.0001$, ** $P \leq 0.01$, * $P \leq 0.05$. (**D**) Immunoblot analysis of TSP-4 in control (ctr) and TSP-4 KO cells. Data are expressed (mean±SD) as residual % of mRNA in KD samples compared to control. $N_{donors} = 5$, one-sample t-test; ** $P \leq 0.01$. (**E**) Flow cytometric analysis (mean±SD) of cytotoxicity mediated by the synaptic output of control (ctr) and TSP-4 KO plated on immobilized ICAM-1 and ICAM-1 + anti-CD3ε mAb. MEC1 B cells, used as targets, were seeded after flushing out the CTLs and incubated for 16 h at 37°C. Targets were then recovered and analyzed by flow cytometry using propidium iodide (PI) to stain dead cells. Quantification (mean±SD) of the target cell lysis (%) (*Left*) and SMAP-mediated cytotoxicity expressed as fold change in KO samples versus ctr. $N_{donors} = 5$, one-way ANOVA test (*Left*) and one-sample t-test (*Right*); ** $P \leq 0.01$, * $P \leq 0.05$, only significant differences are shown. No significant killing was observed at a shorter time of incubation (6 h killing 14.08±1.67% vs 16 h killing 40.1±4.94%).

Figure S11. CTL inhibition by CLL conditioned media correlates with downregulation of *GZMB* and *THBS4* expression. (**A**) Workflow for in vitro generation of CTLs from primary $CD8^+$ T lymphocytes purified from buffy coats of healthy donors in the presence of conditioned media. (**B,C**) Flow cytometric analysis of cell-mediated cytotoxicity by CTLs generated from primary $CD8^+$ T lymphocytes in the presence of media conditioned by either healthy B cells (HD B SN) or B cells purified from CLL patients (CLL B SN), and incubated with SAg-pulsed CFSE-stained MEC1 B cells, used as targets, at the indicated E:T ratios.

Cytotoxicity was assessed by using propidium iodide (PI) uptake by target cells. Quantification (mean±SD) of $CFSE^{+}/PI^{+}$ cells (%) (**B**), representative flow cytometry dot plots (**C**, *Left*) and cell-mediated cytotoxicity expressed as fold change (**C**, *Right*) in samples treated with CLL B SN vs samples treated with HD B SN. $N_{donors} = 3$, two-way ANOVA test (*Left*) and one-sample t-test (*Right*); *** $P \leq 0.001$, ** $P \leq 0.01$, * $P \leq 0.05$, only significant differences are shown. (**D**) RT-qPCR of *GNLY, GZMA, GZMB, PRF, THBS1, THBS* and *SRGN* mRNA in CTLs generated in the presence of media conditioned by either healthy B cells (HD B SN) or B cells purified from CLL patients (CLL B SN). The graph shows the normalized relative abundance of the transcripts (mean±SD, ctr value = 1). $N_{donors} = 6$, one-sample t-test; * $P \leq 0.05$, only significant differences are shown.

Figure S12. Gating strategy used for the flow cytometric analysis of cell-mediated cytotoxicity. (**A**) Raji B cells used as target cells were labeled with CFSE and the analysis was carried out gating on CFSE-positive ($CFSE^{+}$) cells. $CFSE^{+}$ Raji cells were further gated and the analysis of PI^{+} cells restricted to $CFSE^{+}$ cells.

Supplementary tables

Table S1. List of the antibodies used

Fluorophore	Protein target	Clone ID	Supplier and catalogue number	Dilution and application
-	anti-actin	C4	EMD Millipore (#MAB1501)	1:10000 (WB)
FITC	anti-CD45RA	HI100	BioLegend (#304106)	1:50 (FC)
APC	anti-CD62L	DREG-56	BioLegend (#304809)	1:50 (FC)
-	anti-Flag		Cell Signaling (#14793)	1:200 (IF)
-	anti-HA	3F10	Rioche (#45-11867423001**)**	1:500 (IF)
-	anti-GFP	3E6	Invitrogen (#A11120)	1:200 (IF, FC)
-	anti-GFP	-	Invitrogen (#A11122)	1:200 (IF)<br>1:1000 (WB)
-	anti-GM130	35	BD (#610822)	1:100 (IF)
AF647	anti-GzmB	GB11	BioLegend (#515406)	1:20 (IF)
-	anti-GzmB APC	GB11	Invitrogen (#GRB05)	1:200 (IF)
-	anti-LAMP-1 (CD107a)	H4A3	BioLegend (#328602)	1:400 (IF)
AF488	anti-Prf	dG9	BioLegend (#308108)	1:50 (IF)
AF647	anti-Prf	dG9	BioLegend (#308110)	1:50 (IF)
-	anti-RFP	-	Rockland Immunochemicals (#600-401-379)	1:500 (IF, FC)<br>1:1000 (WB)
-	anti-TSP-1	D4.6, A6.1, MBC 200.1	Invitrogen (#MA5-11330)	1:500 (WB)
-	anti-TSP-1	A6.1	Invitrogen (#MA5-13398)	1:50 (IF)
-	anti-TSP-4	F-7	Santa Cruz Biotechnology (#sc-390734)	1:50 (IF)<br>1:500 (WB)
AF488	anti-mouse	-	Invitrogen (#A11001)	1:80 (IF)<br>1:400 (FC)
AF647	anti-mouse	-	Invitrogen (#A21236)	1:80 (IF)
StarGreen	anti-mouse	-	Abberior (#STGREEN-1001)	1:100 (IF)
AF488	anti-rabbit	-	Invitrogen (#A11008)	1:80 (IF)
AF555	anti-rabbit	-	Invitrogen (#A21428)	1:80 (IF)
AF647	anti-rabbit	-	Invitrogen (#A21235)	1:80 (IF)<br>1:400 (FC)
STAR580	anti-rabbit	-	Abberior (#ST580-1002)	1:100 (IF)

StarRed	anti-rabbit	-	Abberior (#STRED-1002)	1:100 (IF)
ATTO647N	anti-rat	-	Biomol (#612-156-120)	1:500 (IF)
Star 568	anti-rat	-	Abberior (#ST580-1007)	1:100 (IF)

Table S2. List of the oligos used

Oligo name	Sequence	Description
GLYN fw	GGATAAGCCCACCCAGAGAAG	RT-PCR primer
GLYN rv	ACAGATCTGCTGGGCAGTTT	RT-PCR primer
GZMA fw	AACCAGGAACCATGTGCCAA	RT-PCR primer
GZMA rv	GGCTTCCAGAATCTCCAT	RT-PCR primer
GZMB fw	TCAAAGAACAGGAGCCGACC	RT-PCR primer
GZMB rv	TTGGCCTTTCTCTCCAGCTG	RT-PCR primer
HPRT-1 fw	AGATGGTCAAGGTCGCAAG	RT-PCR primer
HPRT-1 rv	GTATTCATTATAGTCAAGGGCATATC	RT-PCR primer
PRF fw	CCTGCAGTCACAGCTACACA	RT-PCR primer
PRF rv	GGGGCTCCAGTTAAGGCAAT	RT-PCR primer
SRGN fw	GACGAGAATCCAGGACTT	RT-PCR primer
SRGN rv	GGGCAGATTCCTGTCAAGAG	RT-PCR primer
TSP-1 fw	GCAAGTCACCCAGTCCTACT	RT-PCR primer
TSP-1 rv	AATGAAACCCGTCTTTGGCC	RT-PCR primer
TSP-4 fw	TGCCTGTGATAACTGCCTGA	RT-PCR primer
TSP-4 rv	ATCACCATCCTTGTCCCGTT	RT-PCR primer
18S fw	CGCCGCTAGAGGTGAAATT	RT-PCR primer
18S rv	CTTGGCAAATGCTTTCGC	RT-PCR primer
TSP-1 sgRNA fw	ttaatacgactcactataggCGCCAGACTCCGCATCGC AAgttttagagctagaaatagc	sgRNA production primer
TSP-4 sgRNA fw	ttaatacgactcactataggCTCCGTTACCTGAAGAAC GAGttttagagctagaaatagc	sgRNA production primer
common rv primer	AGCACCGACTCGGTGCCACT	sgRNA production primer
GFPSpark fw	TGCTCTAGAATGGTGAGCAAGGGCGAGGAG	Cloning primer
GFPSpark rv	CCGCTCGAGTTACTTGTACAGCTCGTCCATG	Cloning primer
mCherry fw	TGCTCTAGAATGGTGAGCAAGGGCGAGGAG	Cloning primer
mCherry rv	CCGCTCGAGTTACTTGTACAGCTCGTCCAT	Cloning primer
TSP-4-GFPSpark fw	CGGGGTACCATGCTGGCCCCGCGC	Cloning primer
TSP-4-GFPSpark rv	CCGGAATTCCATTATCGAAGCGGTCGAAATTC TGGGTT	Cloning primer
TSP-4-mCherry fw	CGGGGTACCATGCTGGCCCCGCGC	Cloning primer

TSP-4-mCherry rv	CCGGAATTCCATTATCGAAGCGGTCGAAATTCTGGGT	Cloning primer
TSP-1-mCherry fw	CGGGGTACCATGGGGCTGGCCTGG	Cloning primer
TSP-1-mCherry rv	CCCAAGCTTGGGGATCTCTACATTCGTATTTCAGG	Cloning primer
pMax-TSP-1-linker-3xFLAG fw	ATGTATAGGTACCATGGGGCTGGCCTGG	Cloning primer
pMax-TSP-1-linker-3xFLAG rv	TGCTCTAGAGGGATCTCTACATTCGTATTTCAG	Cloning primer
linker-3xFLAG fw	CTAGAGGTGGGAGCGGCGGAAGCGGCGGTAGCG	Cloning primer
linker-3xFLAG rv	CTAGCGCTACCGCCGCTTCCGCCGCTCCCACCT	Cloning primer
3xFLAG fw 1	CTAGCGACTACAAGGACCACGACGGCGATTATAAGGAT	Cloning primer
3xFLAG fw 2	CACGACATCGACTACAAAGACGACGATGACAAGTAAC	Cloning primer
3xFLAG rv 1	TCGAGTTACTTGTCATCGTCGTCTTTGTA	Cloning primer
3xFLAG rv 2	GTCGATGTCGTGATCCTTATAATCGCCGTCGTGGTCCTTGTAGTCG	Cloning primer
linker-3xHA fw	AATTCAAGGTGGGAGCGGCGGAAGCGGCGGTAGCT	Cloning primer
linker-3xHA rv	CTAGAGCTACCGCCGCTTCCGCCGCTCCCACCTTG	Cloning primer
TSP-4-SBP-mCherry fw	TTGGCGCGCCATGCTGGCCC	Cloning primer
TSP-4-SBP-mCherry rv	CGGAATTCCGATTATCGAAGCGGTCGAAATTCTG	Cloning primer

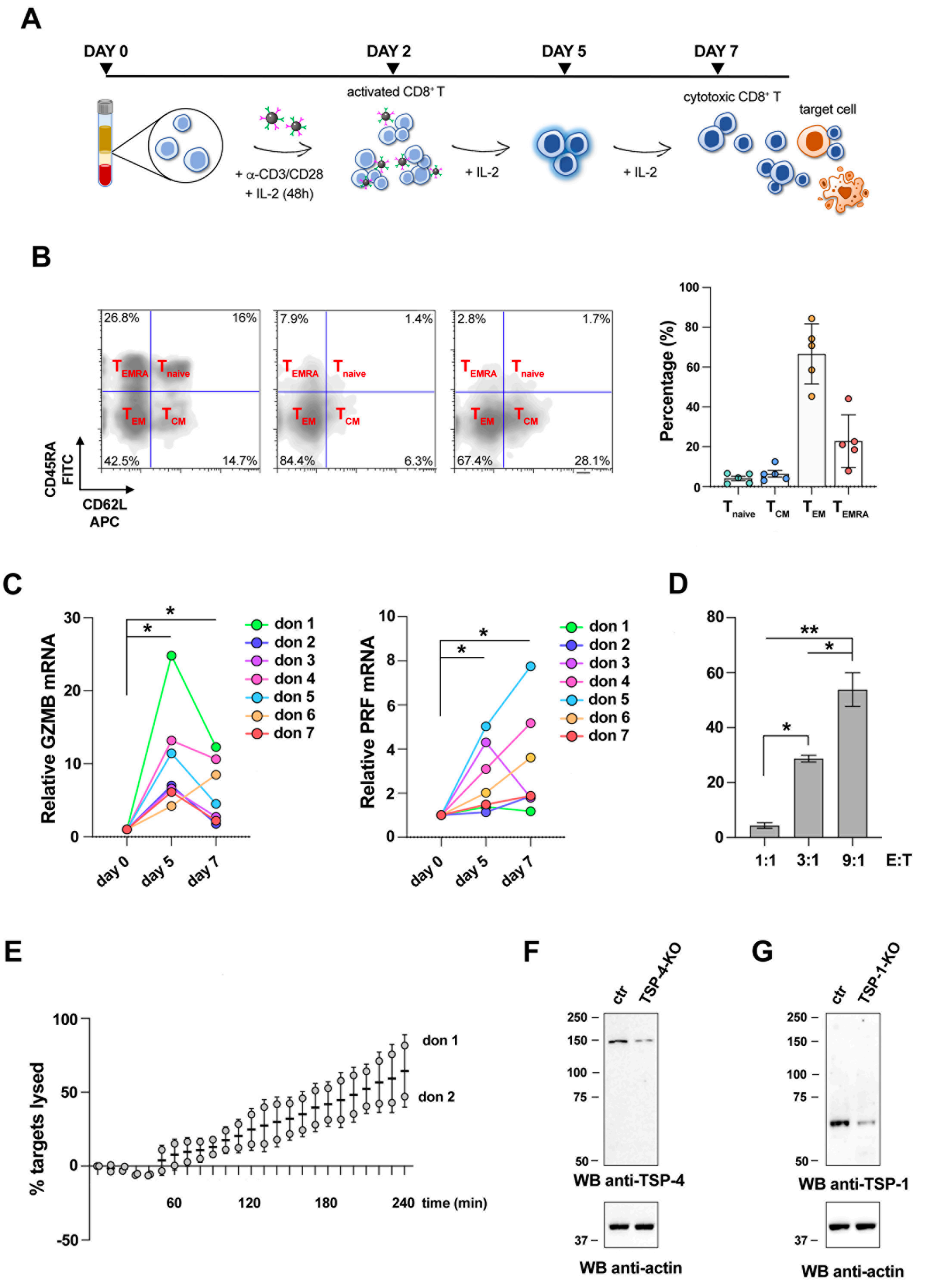
A
DAY 0
DAY 2
DAY 5
DAY 7
activated CD8+ T
cytotoxic CD8+ T
target cell
+ α-CD3/CD28
+ IL-2 (48h)
+ IL-2
+ IL-2
B
26.8%
16%
42.5%
14.7%
7.9%
1.4%
84.4%
6.3%
2.8%
1.7%
67.4%
28.1%
TEMRA
Tnaive
TEM
TCM
CD45RA FITC
CD62L APC
Percentage (%)
Tnaive
TCM
TEM
TEMRA
C
Relative GZMB mRNA
Relative PRF mRNA
day 0
day 5
day 7
don 1
don 2
don 3
don 4
don 5
don 6
don 7
D
1:1
3:1
9:1
E:T
E
% targets lysed
time (min)
don 1
don 2
F
ctr
TSP-4-KO
WB anti-TSP-4
WB anti-actin
G
ctr
TSP-1-KO
WB anti-TSP-1
WB anti-actin

FIGURE S1

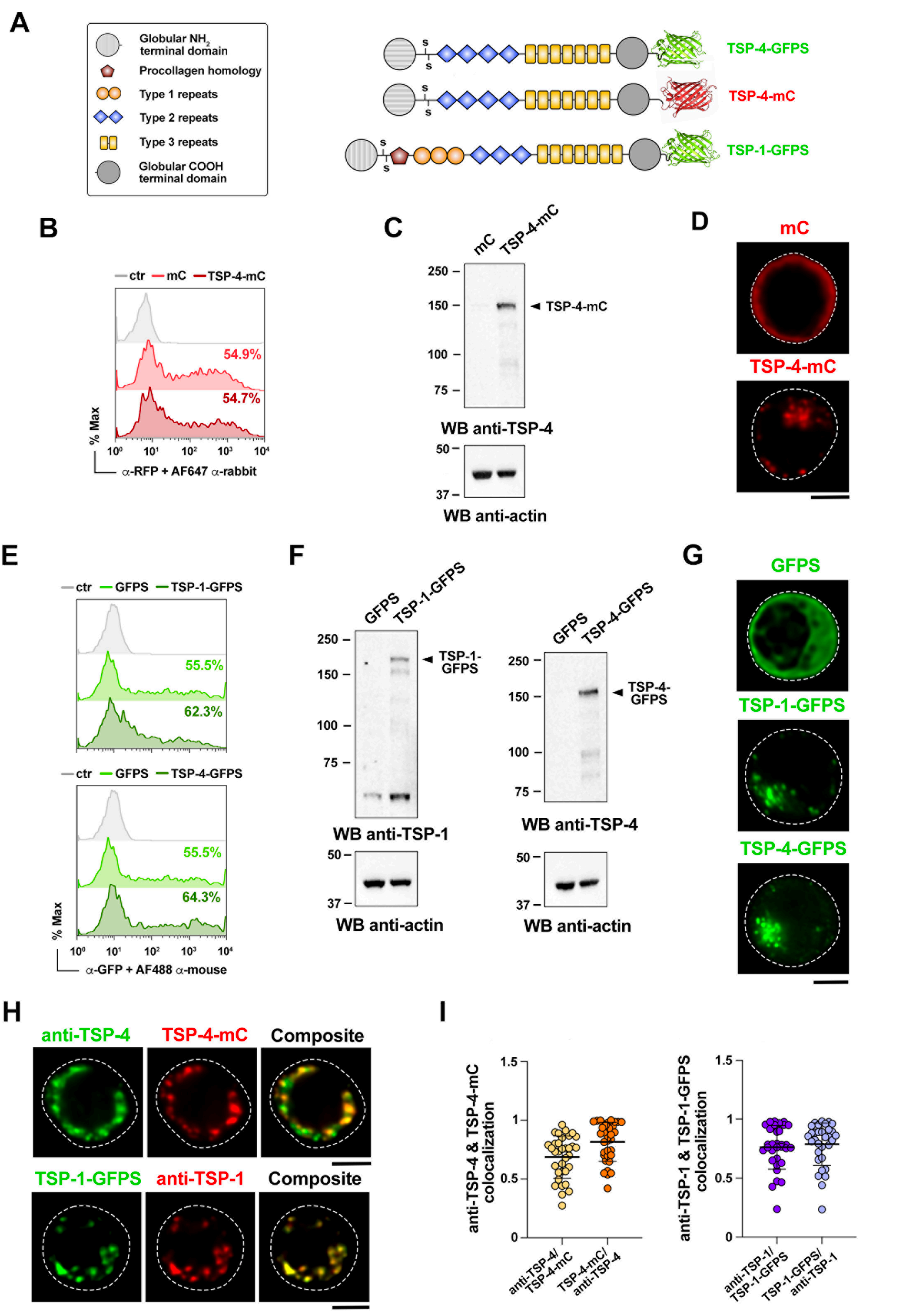
A
Globular NH2 terminal domain
Procollagen homology
Type 1 repeats
Type 2 repeats
Type 3 repeats
Globular COOH terminal domain
TSP-4-GFPS
TSP-4-mC
TSP-1-GFPS
B
ctr mC TSP-4-mC
54.9%
54.7%
% Max
α-RFP + AF647 α-rabbit
C
mC TSP-4-mC
250
150
100
75
TSP-4-mC
WB anti-TSP-4
50
37
WB anti-actin
D
mC
TSP-4-mC
E
ctr GFPS TSP-1-GFPS
55.5%
62.3%
ctr GFPS TSP-4-GFPS
55.5%
64.3%
% Max
α-GFP + AF488 α-mouse
F
GFPS TSP-1-GFPS
TSP-1-GFPS
WB anti-TSP-1
WB anti-actin
GFPS TSP-4-GFPS
TSP-4-GFPS
WB anti-TSP-4
WB anti-actin
G
GFPS
TSP-1-GFPS
TSP-4-GFPS
H
anti-TSP-4
TSP-4-mC
Composite
TSP-1-GFPS
anti-TSP-1
Composite
I
anti-TSP-4 & TSP-4-mC colocalization
anti-TSP-4/TSP-4-mC
TSP-4-mC/anti-TSP-4
anti-TSP-1 & TSP-1-GFPS colocalization
anti-TSP-1/TSP-1-GFPS
TSP-1-GFPS/anti-TSP-1

FIGURE S2

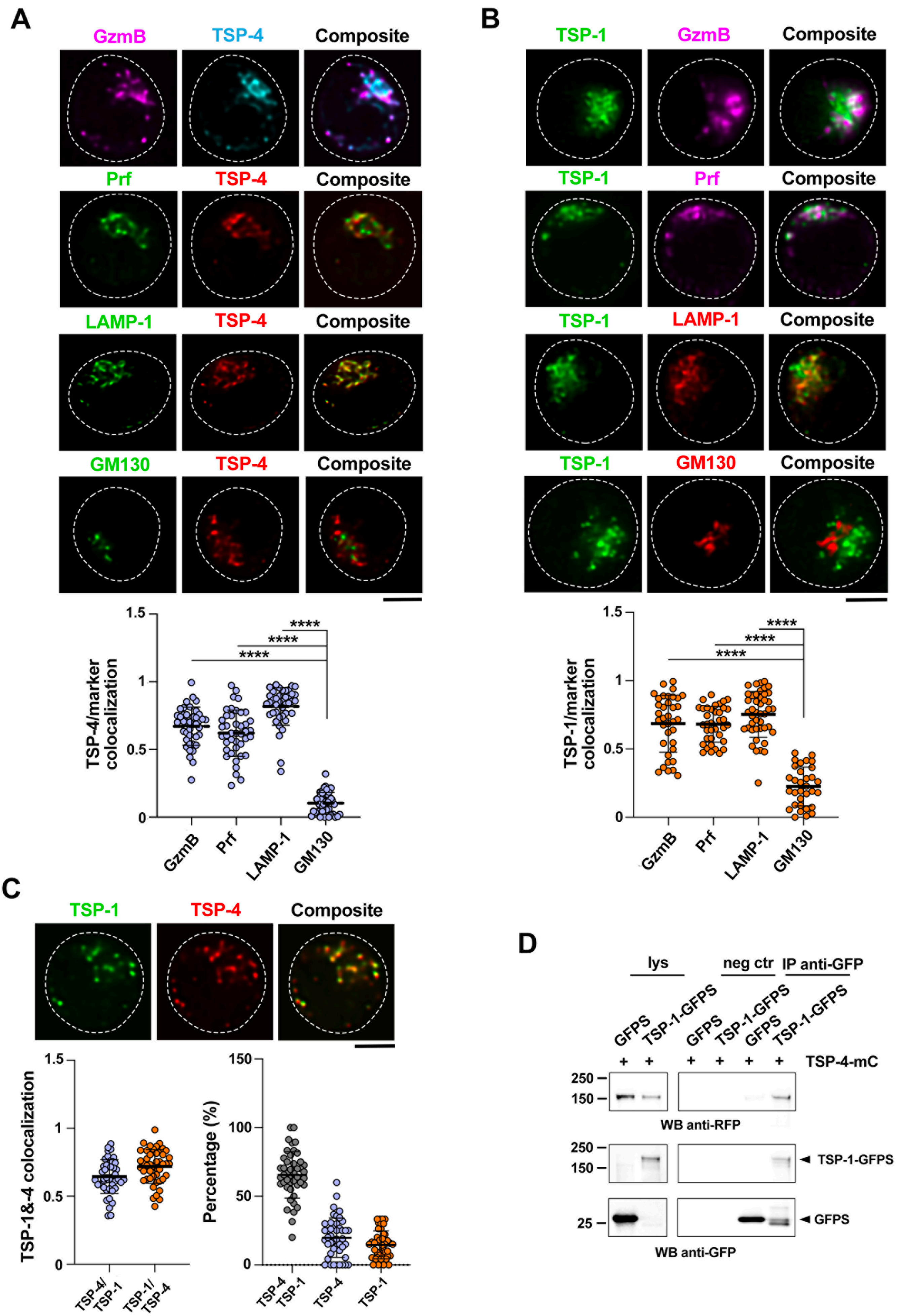
A
GzmB
TSP-4
Composite
Prf
TSP-4
Composite
LAMP-1
TSP-4
Composite
GM130
TSP-4
Composite
TSP-4/marker colocalization
****
GzmB
Prf
LAMP-1
GM130
B
TSP-1
GzmB
Composite
TSP-1
Prf
Composite
TSP-1
LAMP-1
Composite
TSP-1
GM130
Composite
TSP-1/marker colocalization
****
C
TSP-1
TSP-4
Composite
TSP-1&-4 colocalization
TSP-4/TSP-1
TSP-1/TSP-4
Percentage (%)
TSP-4 TSP-1
TSP-4
TSP-1
D
lys
neg ctr
IP anti-GFP
GFPS
TSP-1-GFPS
TSP-4-mC
WB anti-RFP
◄ TSP-1-GFPS
◄ GFPS
WB anti-GFP

FIGURE S3

A

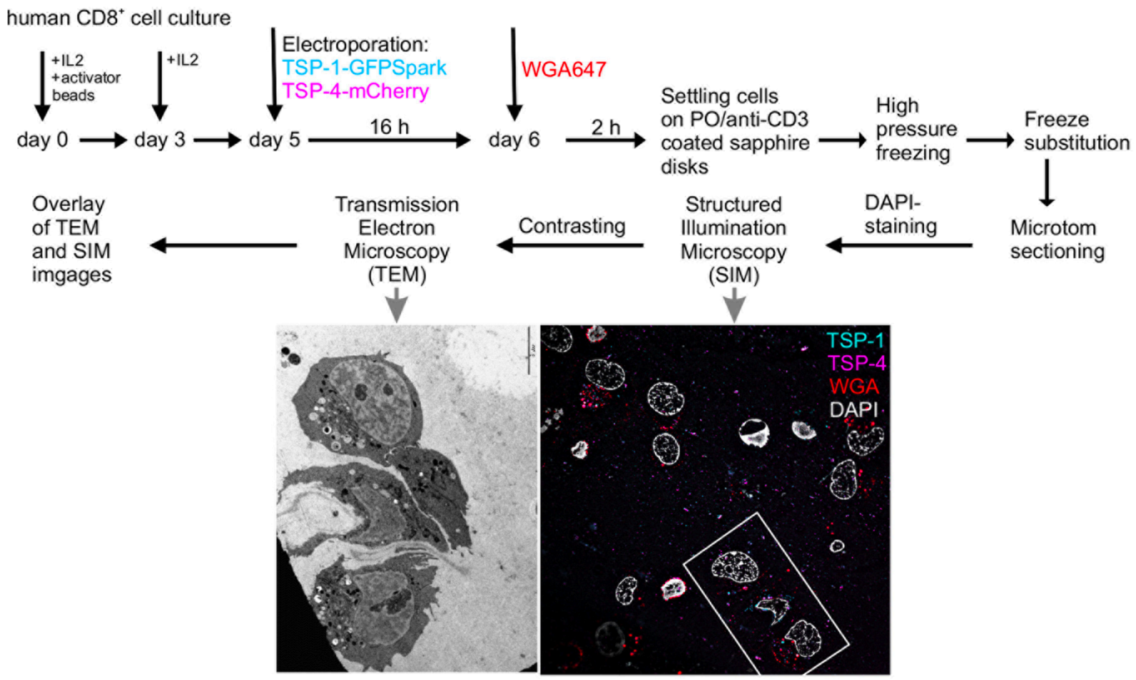
human CD8+ cell culture
+IL2
+activator beads
+IL2
Electroporation:
TSP-1-GFPSpark
TSP-4-mCherry
WGA647
day 0
day 3
day 5
16 h
day 6
2 h
Settling cells on PO/anti-CD3 coated sapphire disks
High pressure freezing
Freeze substitution
Microtom sectioning
DAPI-staining
Structured Illumination Microscopy (SIM)
Contrasting
Transmission Electron Microscopy (TEM)
Overlay of TEM and SIM imgages
TSP-1
TSP-4
WGA
DAPI

B

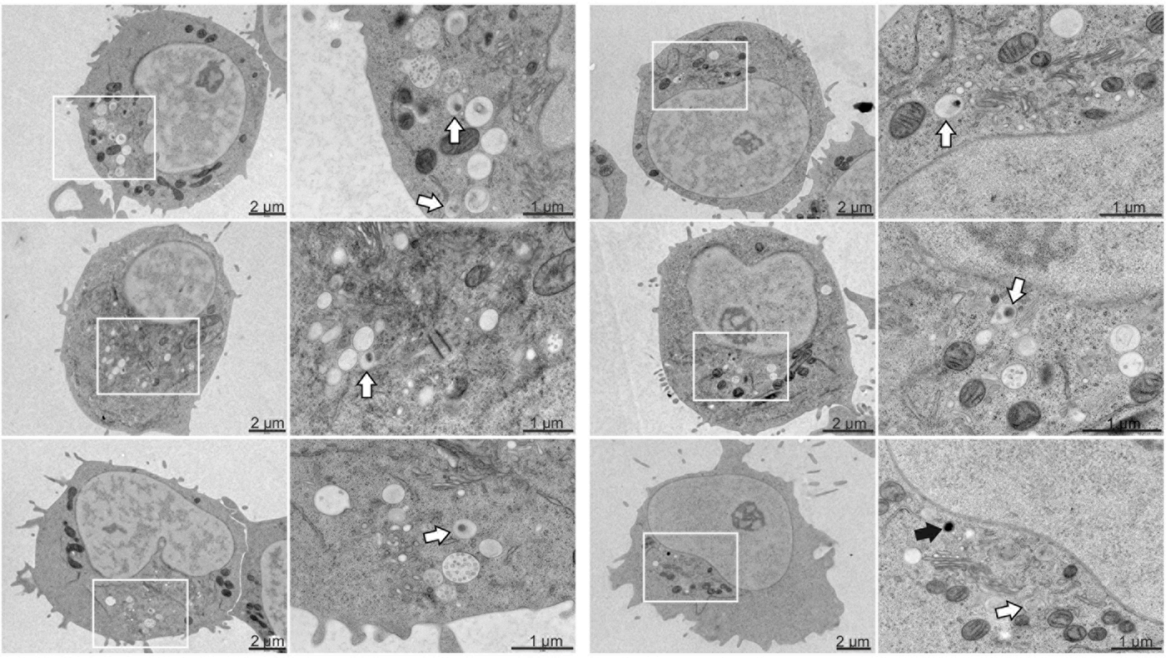
2 µm
1 µm

FIGURE S4

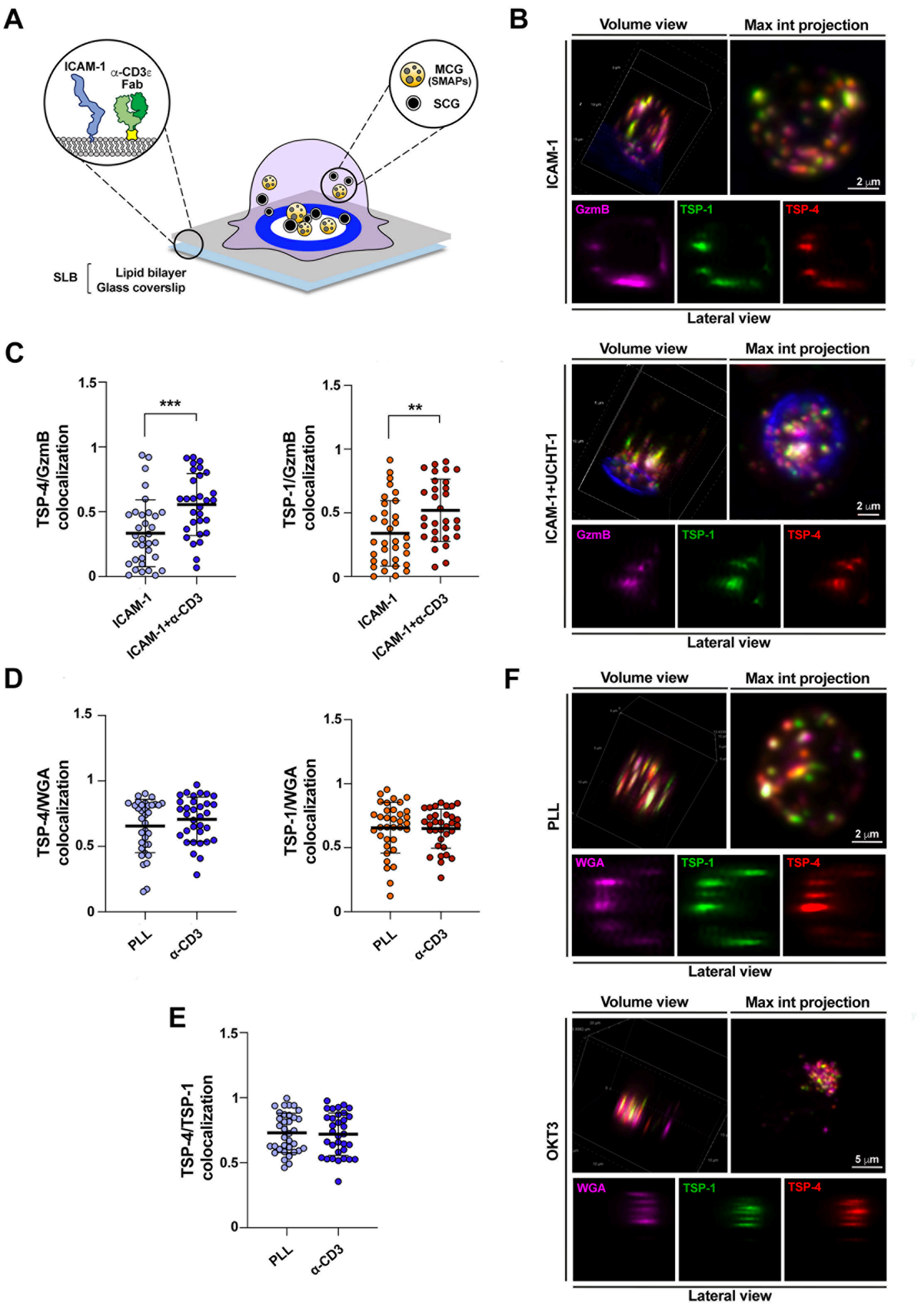
A
ICAM-1
α-CD3ε Fab
MCG (SMAPs)
SCG
SLB
Lipid bilayer
Glass coverslip
B
Volume view
Max int projection
ICAM-1
2 μm
GzmB
TSP-1
TSP-4
Lateral view
ICAM-1+UCHT-1
C
TSP-4/GzmB colocalization
TSP-1/GzmB colocalization
***
**
ICAM-1
ICAM-1+α-CD3
D
TSP-4/WGA colocalization
TSP-1/WGA colocalization
PLL
α-CD3
E
TSP-4/TSP-1 colocalization
F
PLL
WGA
OKT3
5 μm

FIGURE S5

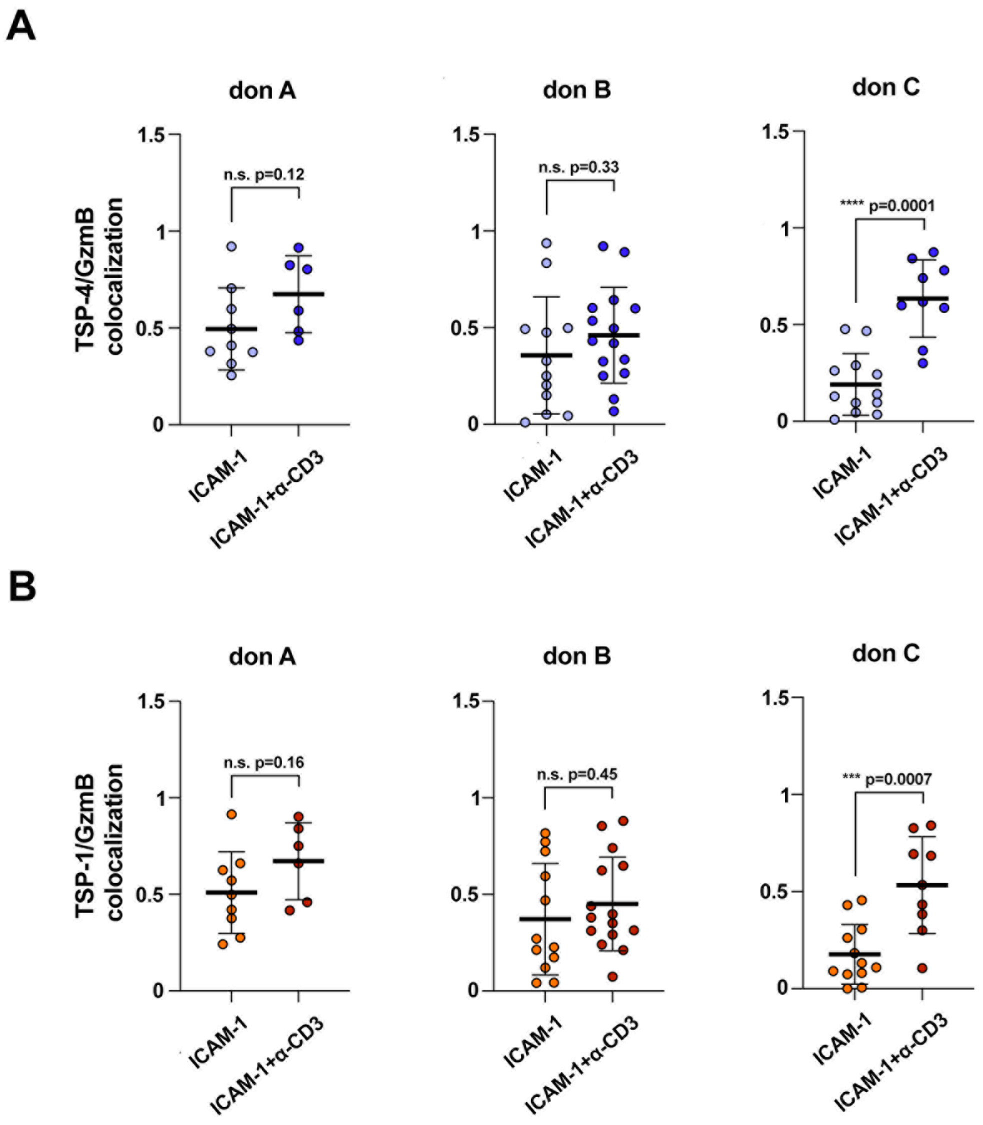
A
don A
don B
don C
TSP-4/GzmB colocalization
n.s. p=0.12
n.s. p=0.33
**** p=0.0001
1.5
1
0.5
0
ICAM-1
ICAM-1+α-CD3
B
TSP-1/GzmB colocalization
n.s. p=0.16
n.s. p=0.45
*** p=0.0007

FIGURE S6

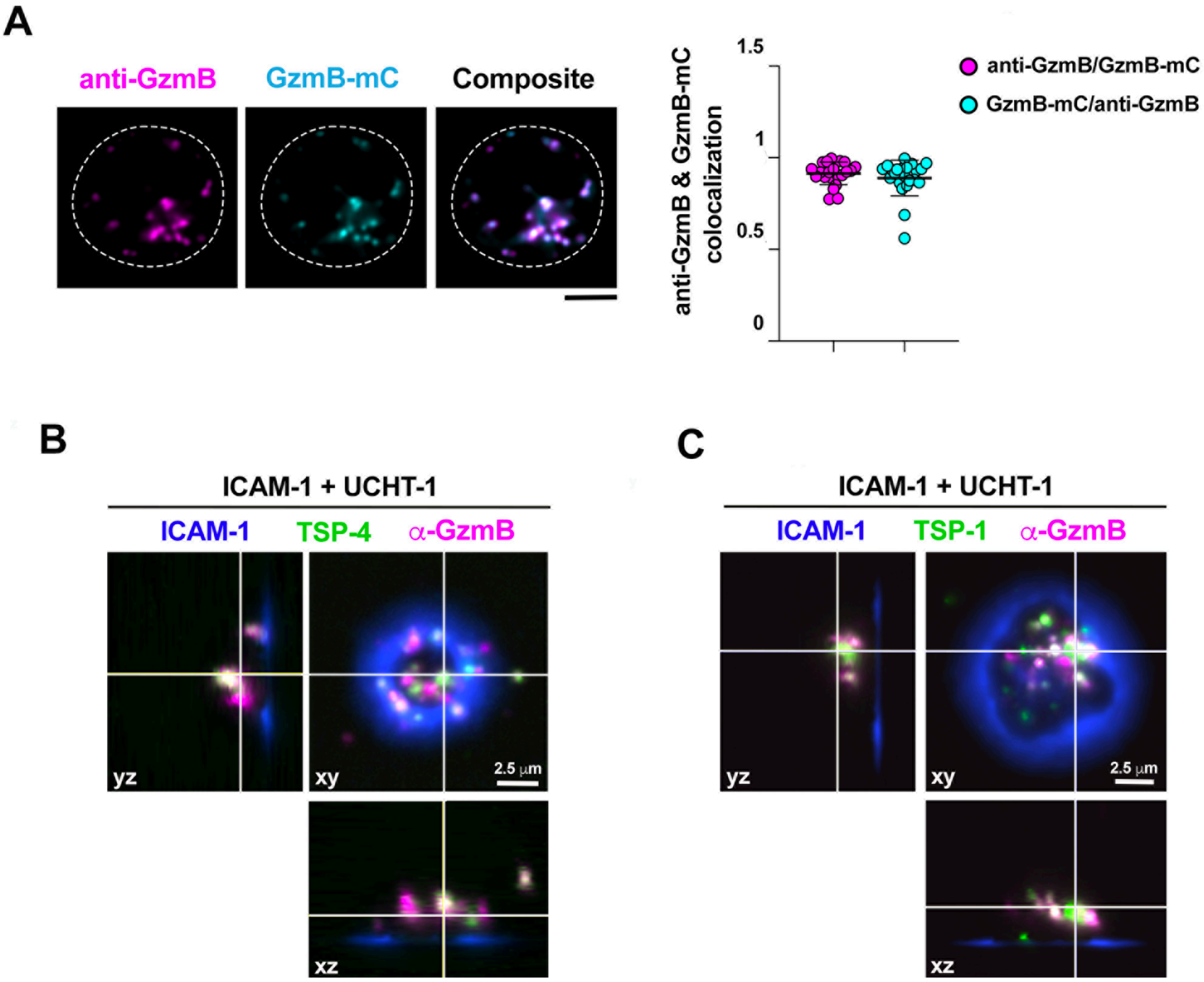
A
anti-GzmB
GzmB-mC
Composite
anti-GzmB & GzmB-mC colocalization
1.5
1
0.5
0
anti-GzmB/GzmB-mC
GzmB-mC/anti-GzmB
B
ICAM-1 + UCHT-1
ICAM-1
TSP-4
α-GzmB
yz
xy
2.5 μm
xz
C
ICAM-1 + UCHT-1
ICAM-1
TSP-1
α-GzmB
yz
xy
2.5 μm
xz

FIGURE S7

A

	Light program		
Lazer	Channel 1 - far red	Channel 0 - red/orange	Channel 2 - blue/green
Fluorophore	WGA-Alexafluor 647	Tsp4-Alexafluor 555	Tsp1-Alexafluor 488
Power	75% lazer power	80% lazer power	80% lazer power
Frames	3000	3000	3000

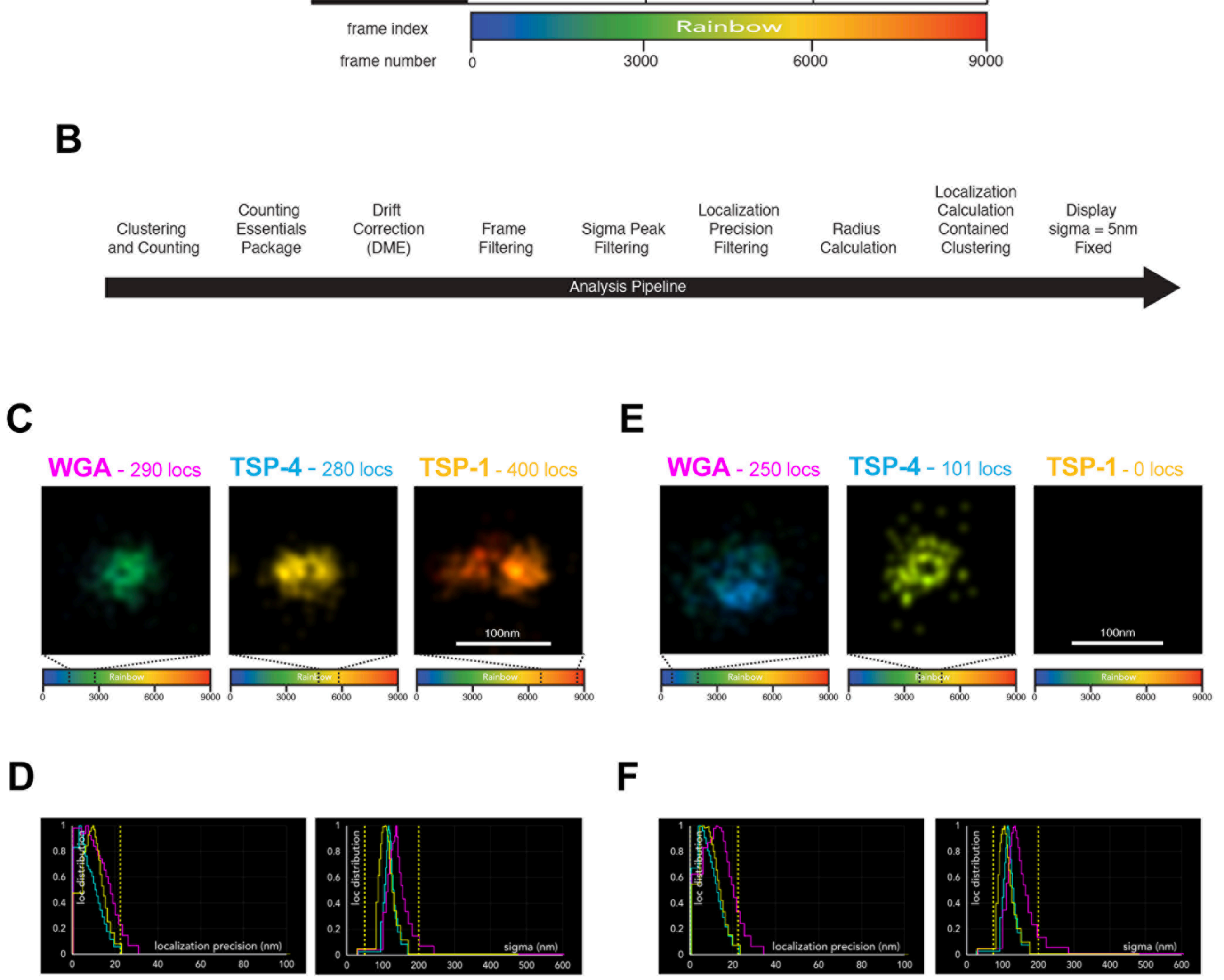

FIGURE S8

A

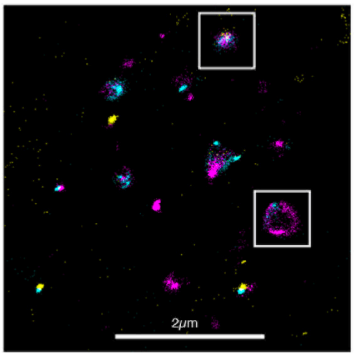

B

	WGA⁺ TSP4⁻ TSP1⁺	WGA⁺ TSP4⁺ TSP1⁻	WGA⁺ TSP4⁺ TSP1⁺
Average Diameter	140nm	181nm	174nm
Number of Particles	9	16	45
Number of Size Outliers	0	0	1

C

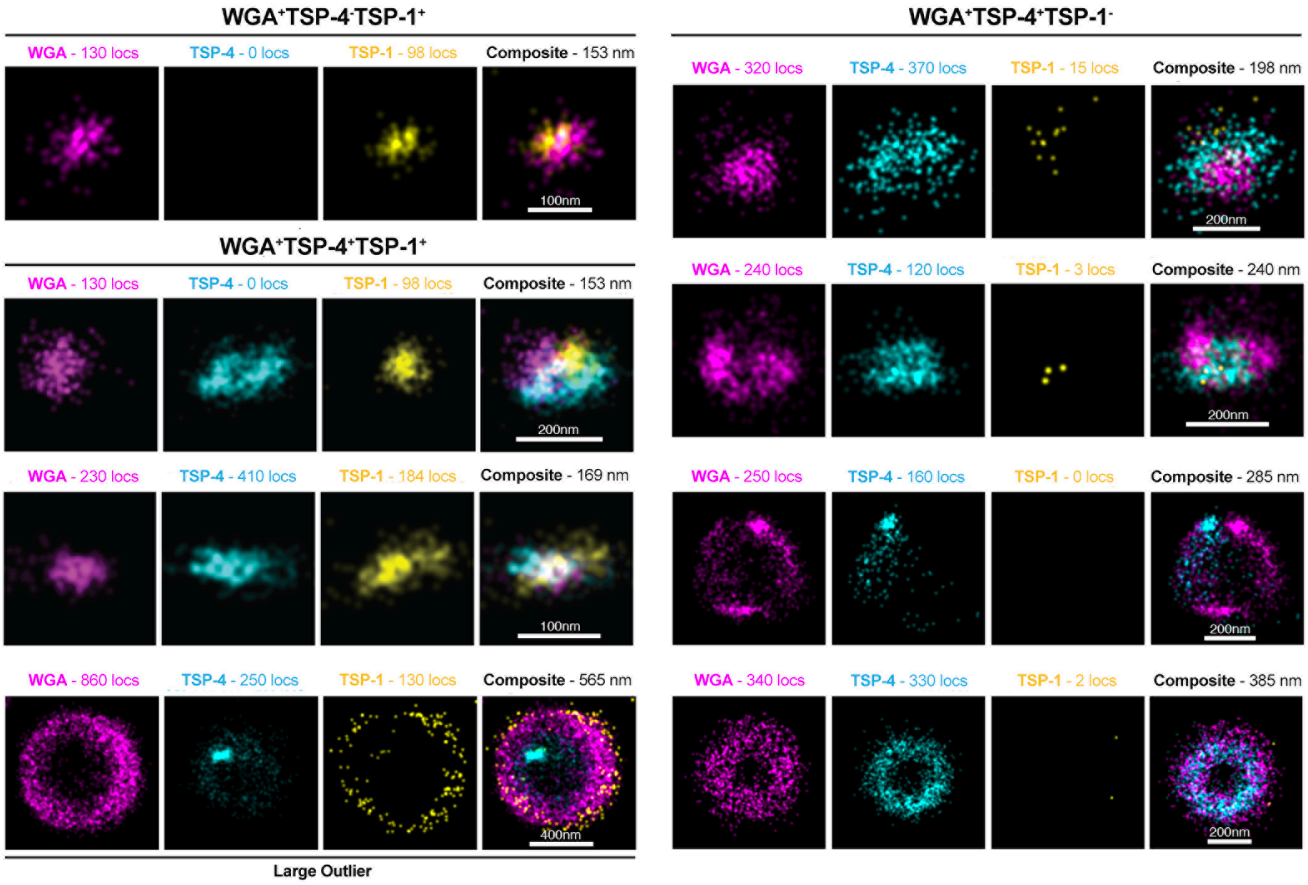

FIGURE S9

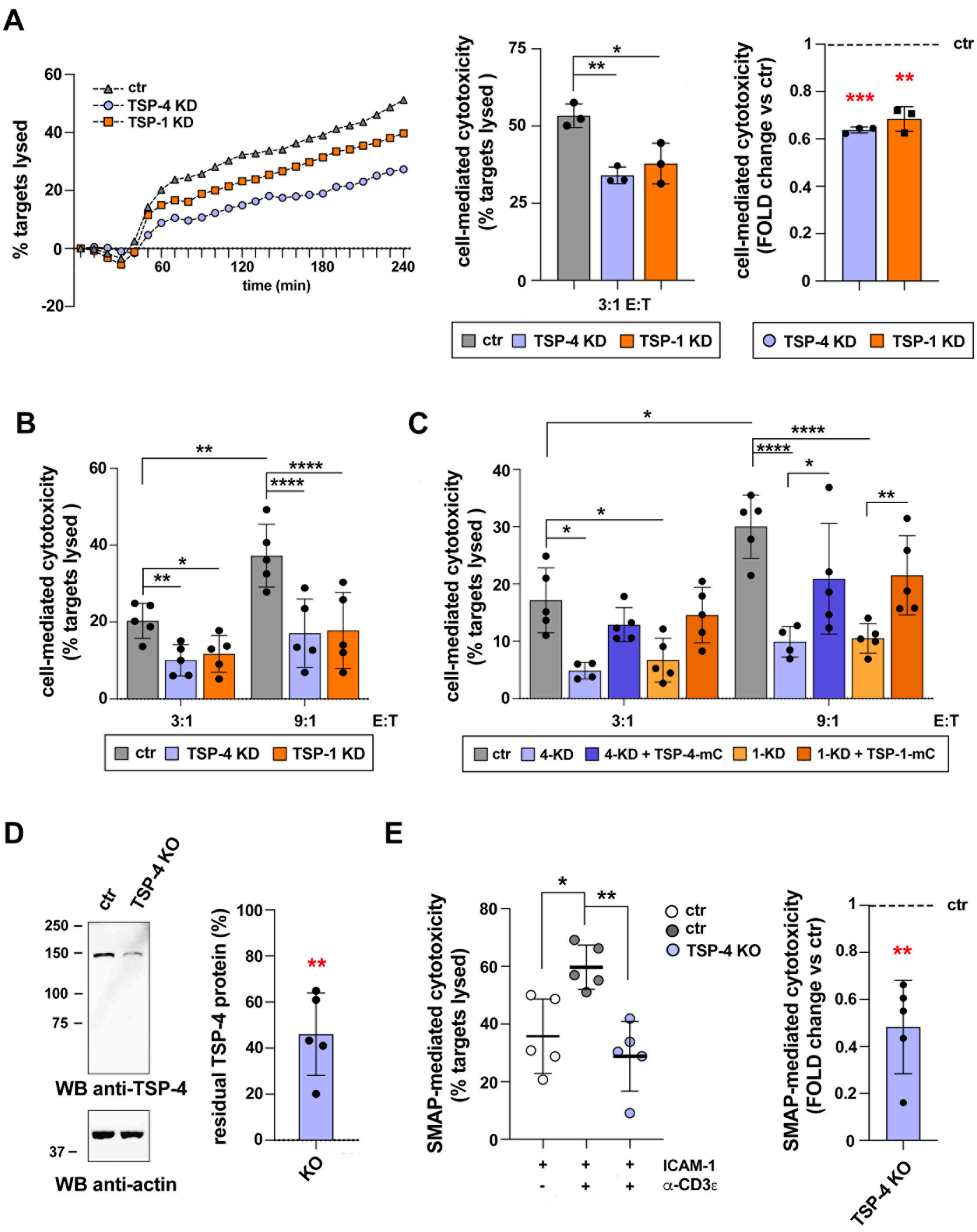
A
% targets lysed
ctr
TSP-4 KD
TSP-1 KD
time (min)
cell-mediated cytotoxicity (% targets lysed)
3:1 E:T
cell-mediated cytotoxicity (FOLD change vs ctr)
B
C
E:T
4-KD
4-KD + TSP-4-mC
1-KD
1-KD + TSP-1-mC
D
WB anti-TSP-4
WB anti-actin
TSP-4 KO
residual TSP-4 protein (%)
KO
E
SMAP-mediated cytotoxicity (% targets lysed)
ICAM-1
α-CD3ε
SMAP-mediated cytotoxicity (FOLD change vs ctr)

FIGURE S10

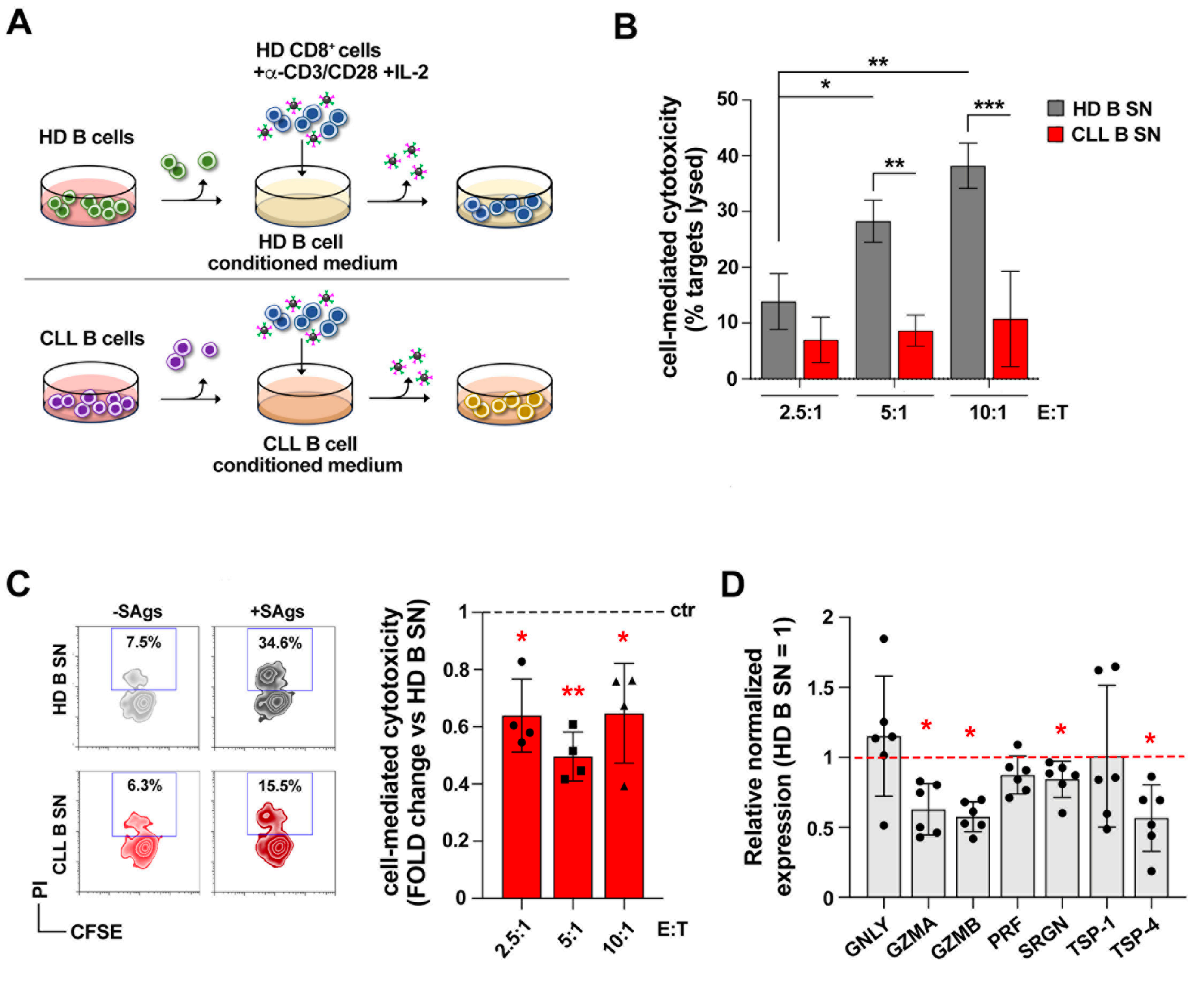
A
HD CD8+ cells
+α-CD3/CD28 +IL-2
HD B cells
HD B cell
conditioned medium
CLL B cells
CLL B cell
conditioned medium
B
cell-mediated cytotoxicity
(% targets lysed)
HD B SN
CLL B SN
2.5:1
5:1
10:1
E:T
C
-SAgs
+SAgs
HD B SN
CLL B SN
7.5%
34.6%
6.3%
15.5%
PI
CFSE
cell-mediated cytotoxicity
(FOLD change vs HD B SN)
ctr
D
Relative normalized
expression (HD B SN = 1)
GNLY
GZMA
GZMB
PRF
SRGN
TSP-1
TSP-4

FIGURE S11

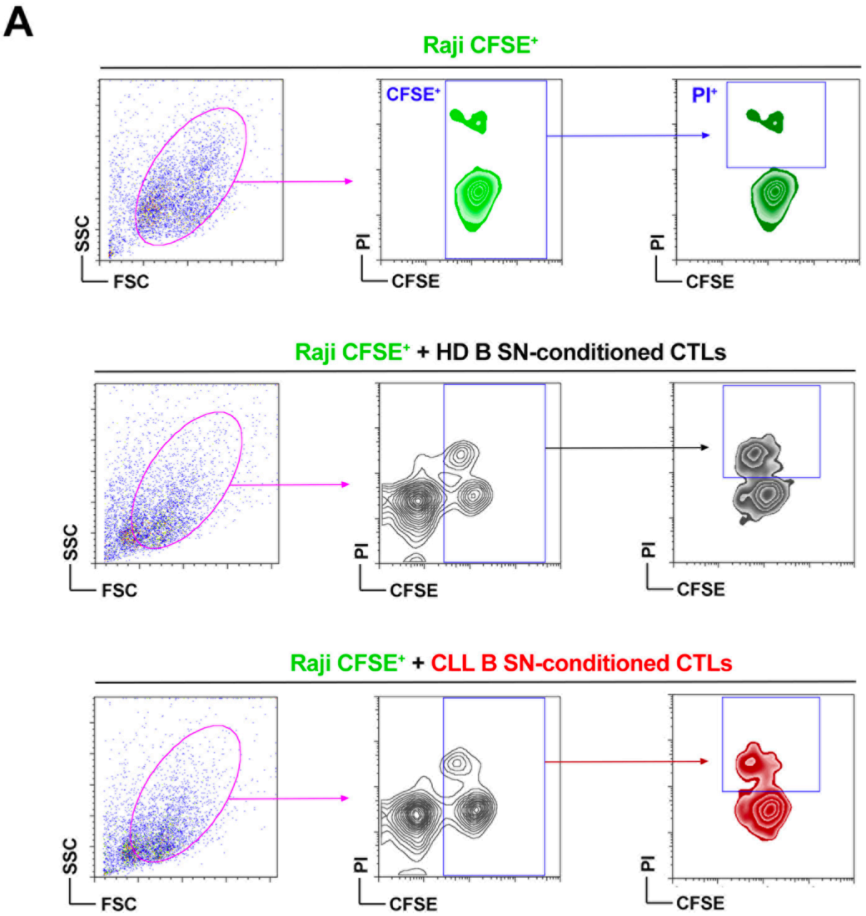
A
Raji CFSE+
CFSE+
PI+
SSC
FSC
PI
CFSE
PI
CFSE
Raji CFSE+ + HD B SN-conditioned CTLs
SSC
FSC
PI
CFSE
PI
CFSE
Raji CFSE+ + CLL B SN-conditioned CTLs
SSC
FSC
PI
CFSE
PI
CFSE

FIGURE S12
